# Structure based design, synthesis and activity studies of small hybrid molecules as HDAC and G9a dual inhibitors

**DOI:** 10.18632/oncotarget.18730

**Published:** 2017-06-28

**Authors:** Lanlan Zang, Shukkoor M. Kondengaden, Qing Zhang, Xiaobo Li, Dilep K. Sigalapalli, Shameer M. Kondengadan, Kenneth Huang, Keqin Kathy Li, Shanshan Li, Zhongying Xiao, Liuqing Wen, Hailiang Zhu, Bathini N. Babu, Lijuan Wang, Fengyuan Che, Peng George Wang

**Affiliations:** ^1^ Central Laboratory, Linyi People's Hospital, Linyi, Shandong 276003, P.R. China; ^2^ Chemistry Department and Center for Diagnostics and Therapeutics, Georgia State University, Atlanta, GA 30303, USA; ^3^ Department of Medicinal Chemistry, National Institute of Pharmaceutical Education and Research (NIPER), Hyderabad, Telangana 500037, India; ^4^ Department of Medicinal Chemistry, National Institute of Pharmaceutical Education and Research (NIPER), Mohali, Punjab 160062, India; ^5^ Department of Immunology, Collaborative Innovation Center of Tianjin for Medical Epigenetics, Tianjin Key Laboratory of Medical Epigenetics, Tianjin Medical University, Tianjin 300070, P.R. China

**Keywords:** epigenetics, pharmacophore, dual inhibitors, G9a inhibitors, HDAC inhibitors

## Abstract

Aberrant enzymatic activities or expression profiles of epigenetic regulations are therapeutic targets for cancers. Among these, histone 3 lysine 9 methylation (H3K9Me2) and global de-acetylation on histone proteins are associated with multiple cancer phenotypes including leukemia, prostatic carcinoma, hepatocellular carcinoma and pulmonary carcinoma. Here, we report the discovery of the first small molecule capable of acting as a dual inhibitor targeting both G9a and HDAC. Our structure based design, synthesis, and screening for the dual activity of the small molecules led to the discovery of compound 14 which displays promising inhibition of both G9a and HDAC in low micro-molar range in cell based assays.

## INTRODUCTION

Epigenetic modifications describe post-translational modifications that occur on the protein without lasting impact on the base genomic code. Likewise, epigenetic modifications are reversible due to the manner in which they occur, making restoration of the epigenome to its normal function a crucial target in many disease models [1–5]. In particular, dimethylation of histone 3 at lysine 9 (H3K9Me2) and various acetylation marks on histones are directly correlated to the onset and advancement of multiple cancer phenotypes, including leukemia, prostatic carcinoma, hepatocellular carcinoma and pulmonary carcinoma [6–8]. Recently, there has been much success in the development of small molecules targeting these post-translational modifications. Epigenetics is still a field much in its infancy considering the number of epigenetic targets [5, 6, 9, 10]. From this vast pool, epigenetic markers that related to leukemogenesis and tumorigenesis has shown to be a promising application of epigenetics.

Cancer is a disease with complicated treatment options due to the multifactorial basis of initiation and progression. Therefore a treatment targeting multiple components instead of a single component could be a particular interest in cancer therapeutics [11–16]. To meet this need, developing new lead molecules which target cancer from various stages of disease development from either known inhibitors or *de novo* is essential for improving effectiveness and side effects/toxicity profile [17–22]. Herein we report the design, synthesis and extensive biological evaluation of a class of small molecules targeting the enzymes histone deacetylases (HDACs) and histone methyltransferases(G9a), both are key posttranslational enzymes in cancer development.

**Figure 1:**
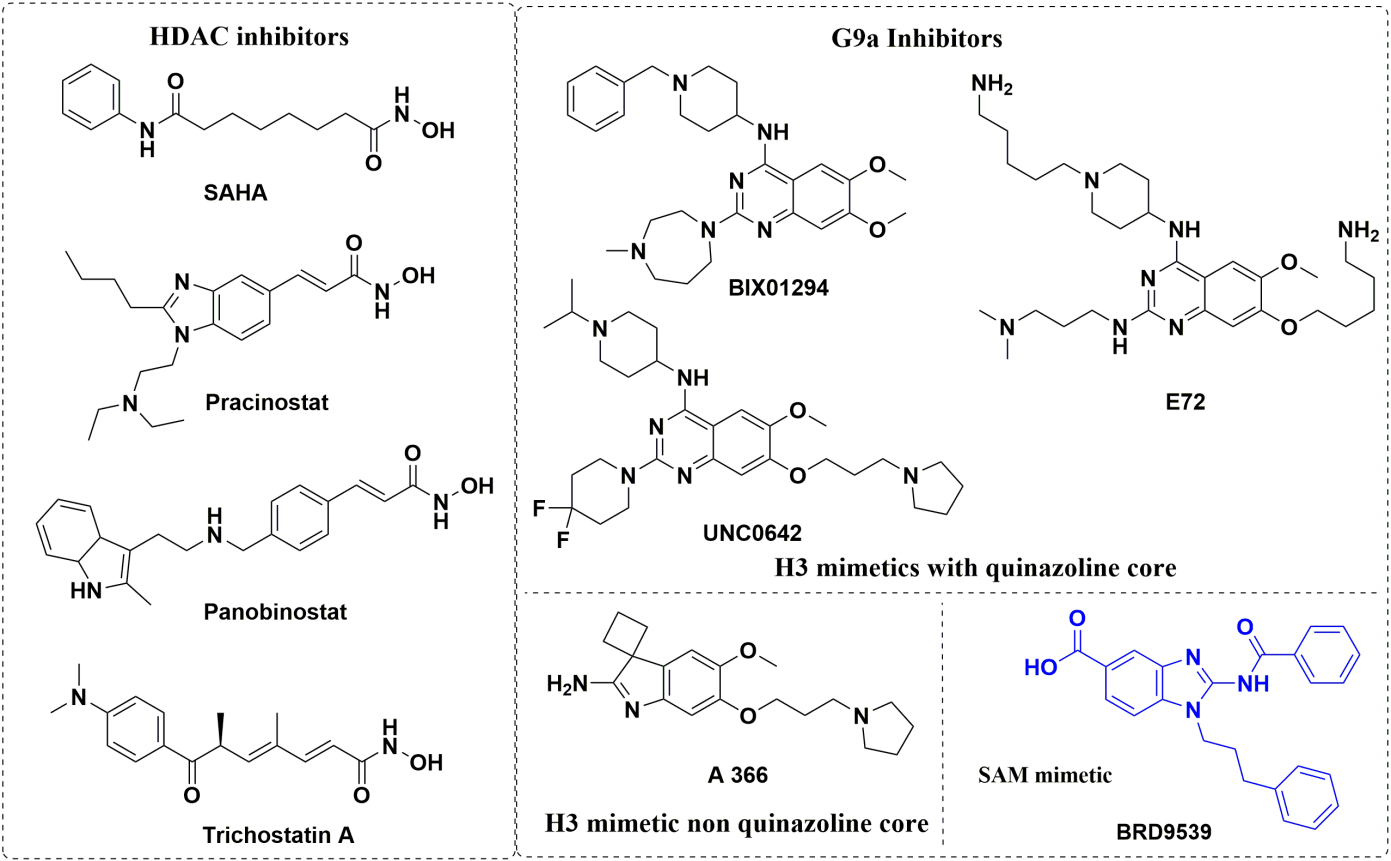
Examples for known HDAC inhibitors and G9a Inhibitors

Histone deacetylases (HDACs) fall into the category of eraser enzymes, so termed due to their ability to reverse the acetylation modification employed by another enzyme histone acetyl transferases (HATs) [23]. However, despite the name, histone deacetylases have a wide range of substrates included but not limited to strictly histones [8]. Aberrant activity of HDACs have been well documented in several cancer phenotypes, with HDAC inhibitors (HDACIs, Figure 1) proven as antineoplastic agents. HDACIs have multiple cell type-specific effects *in vitro* and *in vivo*, such as growth arrest, cell differentiation and apoptosis in malignant cells [6, 24]. HDACIs have been shown to induce apoptosis in both solid and hematopoietic malignancies using both transcription dependent and transcription independent mechanisms [25–27]. While histone acetylation regulated by HDAC and their corresponding writer enzymes HATs, protein methylation is similarly regulated by protein lysine methyltransferases enzymes (PKMT), protein arginine methyltransferases (PRMT) and their corresponding demethylase enzymes [1, 28]. Our particular interest is the PKMT G9a (also known as KMT1C, EHMT2), which is a histone 3 lysine 9 (H3K9) specific methyltransferase that is overexpressed in many cancers including leukemia, hepatocellular carcinoma and pulmonary carcinoma. G9a is notable for its roles in cancer cell proliferation, whereas knockdown of G9a in prostate, lung, and leukemia cancer cells resulted in the inhibition of cell growth [5, 29, 30]. Presently, there are number of small molecules with different structural cores that have been found to inhibit G9a, which are also under consideration in clinical trials [5, 31]. In addition to catalyzing mono- and dimethylation of H3K9, G9a and its closely related protein GLP, also dimethylate lysine 373 of the tumor suppressor p53 to repress the transcriptional activity of p53 [32].

Different modifications of chromatin associated with variable functions, with the extent of modifications and specific residue selected imparting different overall result. For example, hyper-acetylation of histone H3 and H4 both known to be activators associated with ongoing transcription [33, 34]. However, methylation of H3K9 and H3K27 are associated with gene silencing and repressive marks [35]. Following these pathways of distinct and uncorrelated functionalities, we proposed that concurrent inhibition of G9a and HDAC may help histone protein to retain the transcriptional activity via targeting two pathways. First affecting the reduced formation of corepressor marks (H3K9Me2 and p53K373Me2) as a downstream effect of G9a inhibition, and secondly with a relaxed and acetylated chromatin promoted by the HDAC inhibition. We also hypothesize a potential competition between the HDAC and G9a enzyme because they share common substrate (H3K9 and p53K373), a substrate in its acetylated state cannot be further methylated, thereby causing an indirect inhibition of G9a. It should also be noted that HDACIs show only a moderate and limited biological response when applied as a mono treatment, with therapeutic effect significantly improved in combination with other anticancer agents [17, 19, 36]. Hence, the optimal deployment of these molecules may be a combination with other epigenetic drugs, acting against the set of enzymes responsible for the setup and maintenance of epigenetic information [37].

## RESULTS AND DISCUSSION

Initial tests designed around assessing whether a synergistic effect would be observed in real when a combination of G9a inhibitor and HDAC inhibitor used in conjunction. Towards this, the MDA-MB-231 and MCF-7 cell lines treated with either SAHA (1-100 μM), BIX-01294 (1-100 μM), or a mixture of SAHA and BIX-01294 (1:1; 1-100 μM). As indicated in Table 1 (EC_50_ graphs available in Supplementary Information, Supplementary Figure 1), when applied in combination performance was enhanced towards MDA-MB-231 and comparable in MCF-7. Despite being two distinct molecules with different physiochemical properties, application of both displayed a significant improvement (approximately 34% lower EC_50_ to SAHA, and 13% lower EC_50_ to BIX-01294 in MDA-MB-231). Effectively, this provided the basis for incorporating both SAHA and BIX-01294 into a single moiety capable of preserving inhibitory activity against both targets.

**Table 1:** Combination study of BIX-01294 and SAHA

Inhibitor (10 μM)	MDA-MB-231	MCF-7
SAHA	2.874±0.84	8.124±4.98
BIX-01294	2.155±0.88	8.103±1.99
SAHA+BIX-01294	1.891±0.56	8.564±2.17

Multi-target treatments are typically utilized two approaches, formulating two active moieties as a cocktail, or hybridizing properly selected active moieties into a single molecule. The first method heavily relies on both compounds having comparable solubility, or else fine-tuning the formulation to ensure the desired bioavailability [11, 14, 20, 38, 39]. In contrast, a dual-target drug doesn't have such issue with different solubility or bioavailability. However, hybrid compounds are challenging to design due to the difficulties in optimizing a pharmacophore from two dissimilar compounds that can retain multiple functionalities inside the body. There have been some notable successes in the latter regard, with few drugs already on the market with superior performance to their cocktail counterparts [21]. Currently available hybrid drugs target different stages of disease development despite their precursors targeting the same diseases [17, 20, 39, 40]. In light of these studies, we propose that a hybrid drug that instead targets components belonging to the same scheme in disease progression or has otherwise interdependent functionality would yield an improved synergistic effect. Herein we report the design, synthesis, screening and biological study of a lead molecule for further development as a drug candidate utilizing this principle.

### Compound design and synthesis

**Figure 2:**
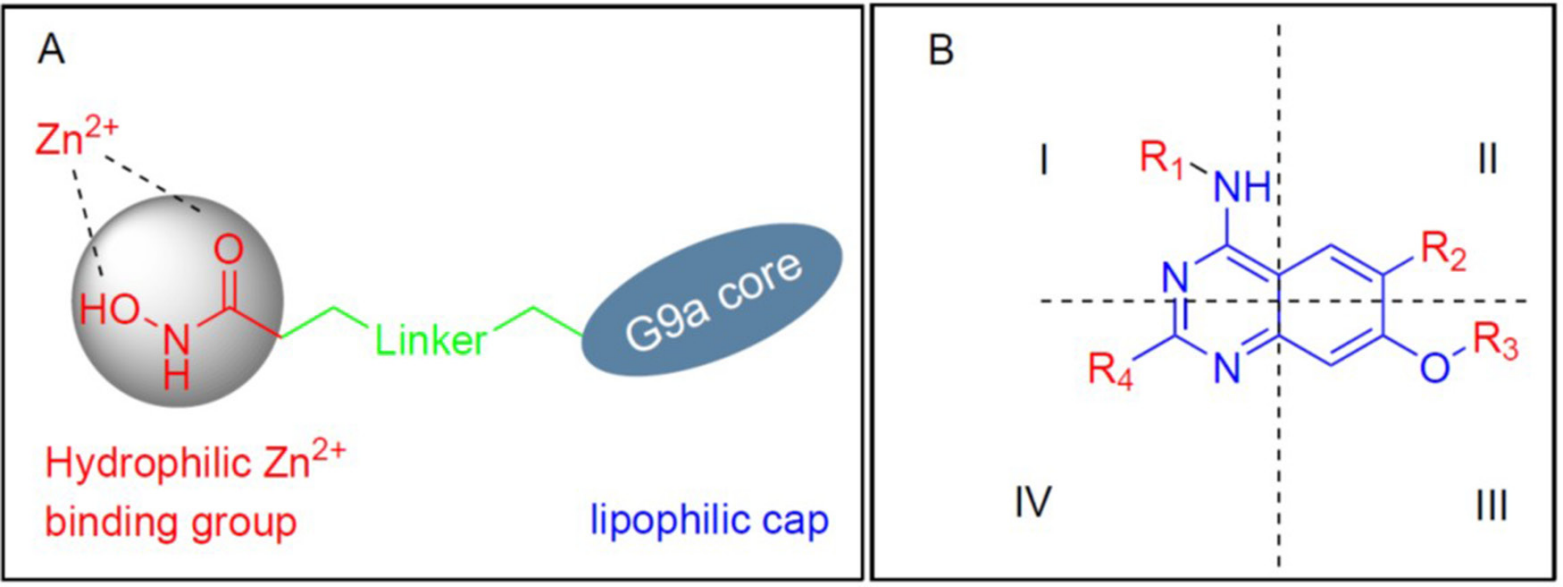
Diagram for Drug design **(A)** Design for Hybrid Molecules, **(B)** Structural representation of designed compounds (R = Linker + Hydroxamic acid).

From the available data on G9a and HDAC in the forms of known inhibitors and their respective X–ray crystal structures, we observed several details regarding their prospective ligands. For instance, all HDAC inhibitors are comprised of three parts- a lipophilic cap connected to a hydrophilic Zn^2+^ binding group by an alkyl, or arylene linker, with the cap portion being different bulky groups. Likewise, current G9a inhibitors, except for the fungal metabolite chaetocin, are primarily derived from the parent compound BIX-01294. Since the discovery of BIX-01294, a clear majority of the G9a inhibitors to emerge based on the quinazoline core of BIX-01294. As the lipophilic quinazoline core resemble the lipophilic bulky cap for HDAC inhibitors, we reasoned that the G9a core could feasibly function as the core scaffold of a HDAC and G9a dual inhibitor. There have been notable success stories in regard of varying the cap or linker portion of the HDAC inhibitor, while the metal binding portion kept as a hydroxamic acid or an *ortho*-amino benzamide [11, 19, 21, 41, 42]. Following this line of thinking, we added the linker and the hydroxamic acid at the C2, and C4 of quinazoline ring to obtain the desired hybrid molecules (Figure 2A). This design was inspired by the fact G9a have many inhibitors with bulky side chains as in the case of E72, HDACIs can also afford a reasonable variety of lipophilic cores (Figure 1). Various analogs with different linker lengths and diverse groups at C6 and C4 cyclohexylamine positions were also designed. All these compounds classified into three for ease of reference.

### Synthesis

**Figure 3:**
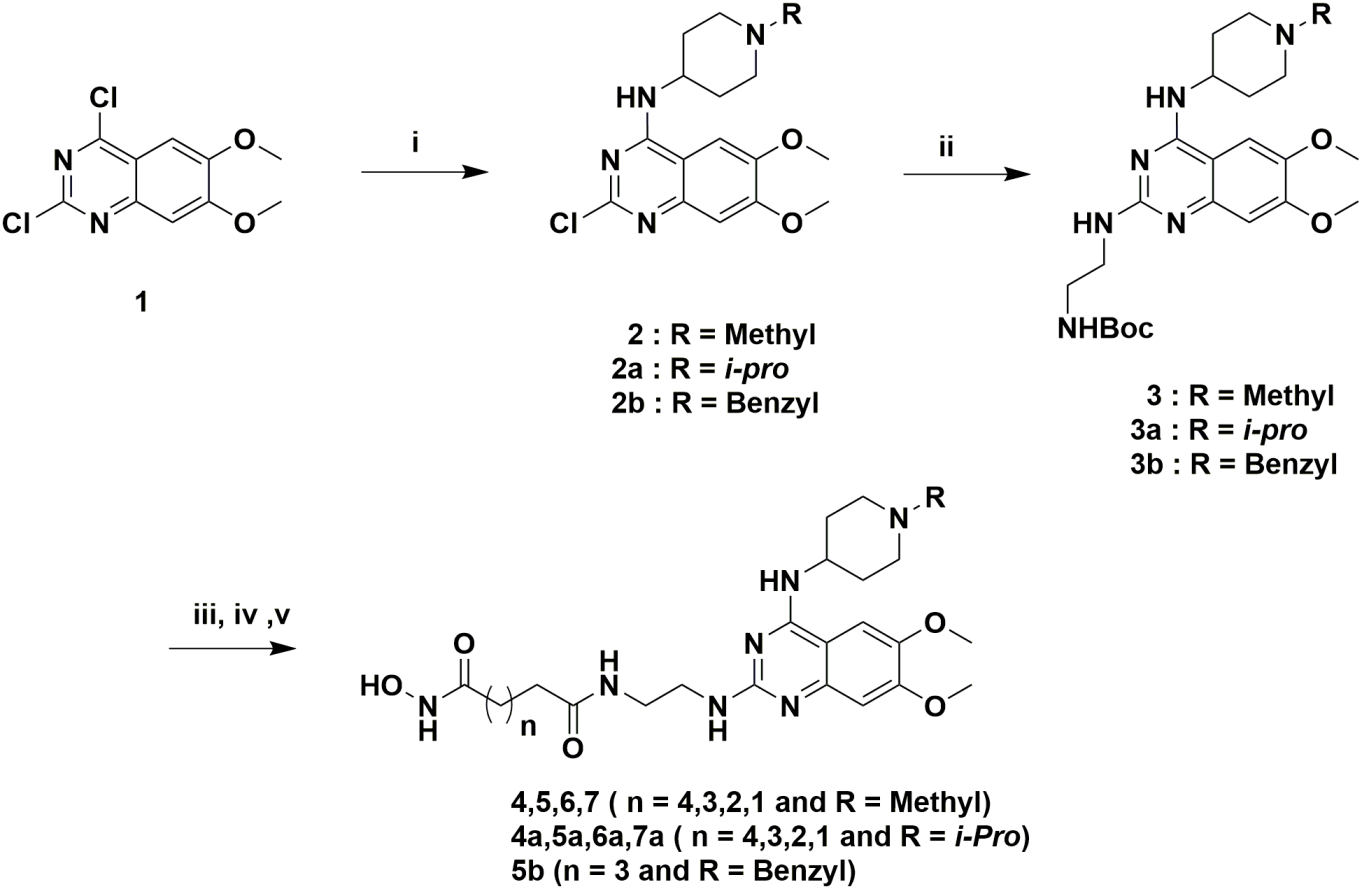
Reagents and conditions for scheme 1 **(i)** 1-methylpiperidin-4-amine/1-isopropylpiperidin-4-amine, DIPEA, DMF, rt, 3 h, 80-86%, **(ii)** tert-butyl (2-aminoethyl)carbamate, DIPEA, 160°CMicrowave, 10 min, 60-66%, **(iii)** TFA/DCM, 3 h, **(iv)** MonomethylSuberate, EDCl, HOBt, 8 h, **(v)** 50% NH2OH in water, MeOH, 60°C, 8 h, 30-38% over two steps.

**Figure 4:**
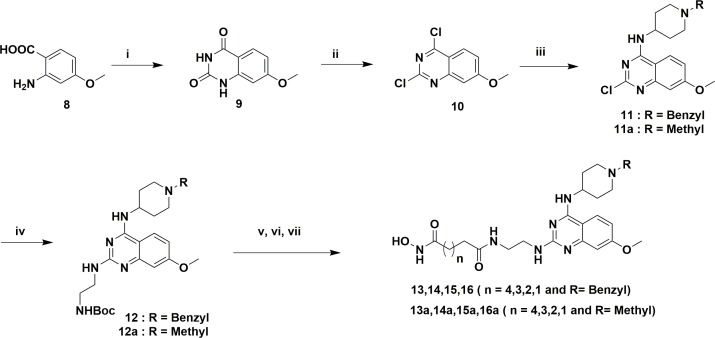
Reagents and conditions for scheme 2 **(i)** Urea, 200°C, 2 h, **(ii)** POCl3, reflux 16 h, 40% in two steps, **(iii)** 4-aminobenzylpiperidin/1-isopropylpiperidin-4-amine, DIPEA, DMF, rt, 3 h, 74% and 86%, **(iv)** tert-butyl (2-aminoethyl)carbamate, DIPEA, 160°C Microwave, 10 min, 64% and 68%, **(v)** TFA/DCM, 3 h, **(vi)** MonomethylSuberate, EDCl, HOBt, 8 h, 70% in two steps, **(vii)** 50% NH_2_OH in water, MeOH, 60°C, 8 h, 30-40%.

The designed analogs were synthesized from two building blocks; commercially available 2,4-dichloro-6,7-dimethoxyquinazoline (**1**) was used for the dimethoxyanalogs (Figure 3) and, 2-amino-4-methoxybenzoic acid (**8**) used for monomethoxyanalogs (Figure 4). Initially, we synthesized only a few analogs of class III to assess the effectiveness of the HDAC substitution while opening the piperazine ring originally present at the prototype BIX-01294, this class was particularly intended to explore the SAR of R1. The bulky seven-member ring was then replaced with an ethylene diamine and coupled with various esters of different chain lengths (three to seven carbons for investigating the optimal length for the HDAC inhibition) to produce compounds **4, 5, 6** and **7**. To check the effect of bulky groups at the C4 position of the heterocyclic ring, an isopropyl group then introduced to the tertiary amine instead of the methyl group to produce the set of compounds **4a, 5a, 6a** and **7a** (Series IIIA, Figure 3). While investigating the binding characteristics of known G9a/GLP inhibitors, the C6 methoxy group of quinazoline ring was hypothesized not to contribute significantly to ligand-receptor interactions. Therefore, the methoxy at C6 was eliminated to find a balance for HDAC activity. Compounds in class II designed from this rationale. Compounds **13-17** have a 4-aminobenzyl piperidine at C4, while **13a-17a** possess an isopropylpiperidin-4-amine. Recent studies of UNC0965 wherein a biotin tag was applied to UNC0638 found that UNC0965 retained biological activity [43], following this lead, C4 of quinazoline ring was explored. Compounds with the HDAC pharmacophore on the C4 carbon of quinazoline core termed as class I, with analogs**19** and **20** retaining the C6 methoxy group, and **21** and **22** lacking the methoxy group.

**Figure 5:**
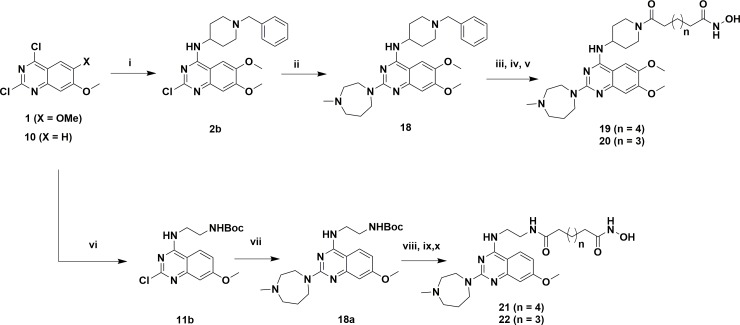
Reagents and conditions for scheme 3 **(i)** 4-aminobenzylpiperidin, DIPEA, DMF, rt, 3 h, 90%, (ii) 1-methyl-1,4-diazepane, DIPEA, 160°C Microwave, 10 min, 74%, **(iii)** EtOH, Pd/C, H2, 8 h, **(iv)** Monomethylsuberate/monomethylpimelate, EDCl, HOBt, 8 h, **(v)** 50% NH2OH in Water, MeOH, 60°C, 8 h, 44% and 45%, **(vi)** NHBoc-ethylinediamine, DIPEA, DMF, rt, 3 h, 78%, **(vii)** 1-methyl-1,4-diazepane, DIPEA, 160°C Microwave, 10 min, 69%, **(viii)** TFA/DCM, 8 h, **(ix)** Monomethylsuberate/ monomethylpimelate, EDCl, HOBt, 8 h, **(x)** 50% NH2OH in water, MeOH, 60°C, 8 h, 29%-36%.

Figure 3 compounds were synthesized from the commercially available starting material **1**. An initial displacement reaction using a primary amine was used to introduce the C4 selective substitution, with the second displacement to introduce the linker at the C2 position following the microwave assisted reaction previously reported [44]. Boc-protected ethylene diamine was treated with compound **2** at 160°C in a microwave for 10 min to yield product **3** with excellent yield. Afterward, the amine 3 was deprotected with TFA/DCM, and the free amine was treated with corresponding monomethyl esters (carbon chain 2-6) in the presence of coupling reagent EDCI and HOBt for about 8 hours to produce mono methyl ester substituted at the C2 position. The ester compounds further treated with hydroxylamine in water to get the corresponding hydroxamic acid derivatives, which were purified using reverse phase flash chromatography to obtain compounds **4-7** and **4a-7a** in good yield. Synthesis of compounds in Figure 4 requires the C6 demethoxy core, and this was synthesized according to the reported procedure to yield **10** [45]. Appropriate displacement and coupling reactions in this core as demonstrated in Figure 4 afforded compounds **13-17** and **13a-17a**. Compounds with the HDAC pharmacophore at the C4 position (Figure 2B) were synthesized from the starting material **1**. As Figure 5 showed, Pd/C hydragenolysis was used to eliminate the benzyl group and produce the free amine of **18** for the coupling of monomethyl esters to result in compounds **19-22**. A parallel synthesis and testing strategy were used in establishing the primary SAR, with the clear rationalization of the structure and activity at each stage we could reduce the synthetic targets. Initially, we evaluated the G9a potential after the introduction of HDAC pharmacophore. A biochemical assay using MALDI-TOF was used to visualize the effects of the synthesized compounds on G9a enzymatic activity, we carried out a biochemical reaction involving target enzyme G9a, methyl donor SAM and substrate H3 peptide at a concentration of 400 nM, 10 μM and 5 μM respectively [46]. After successfully optimizing the reaction conditions and reaction time to see at least 80% of the substrate converted to the methylated form (H3K9Me1 or H3K9Me2) with no tri-methylation, we tested BIX-01294 for an optimum level of inhibition and fixed the concentration as 5 μM for each inhibitor.

**Figure 6:**
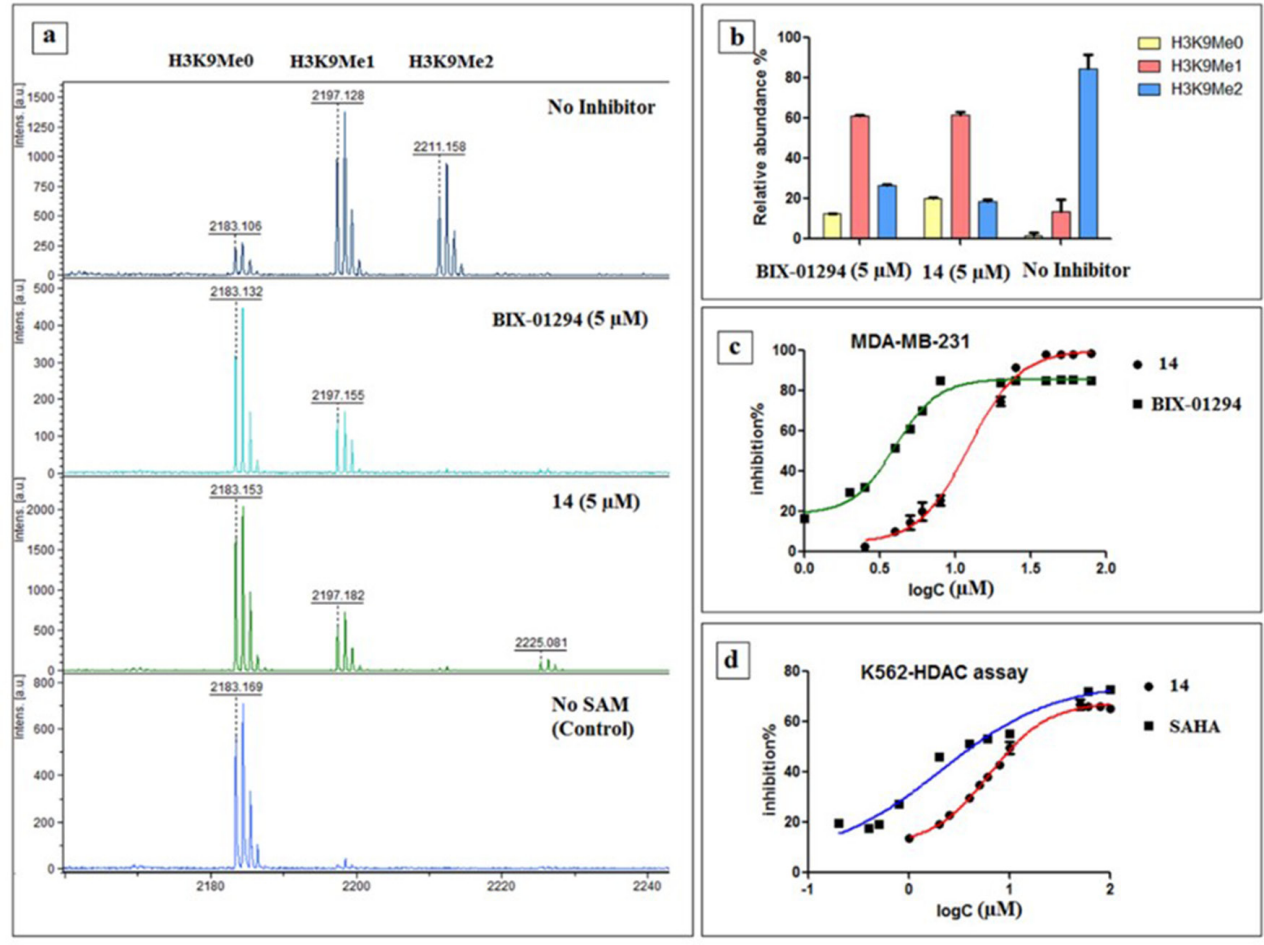
Results of biological assays **(a)** Methylation pattern observed via MALDI-TOF after incubating with inhibitor 14 and BIX-01294 for 30 min, **(b)** % ratio of the H3K9Me0, H3K9Me1 and H3K9Me2 after incubating 30 min with 14 and BIX-01294 versus no inhibitor, **(c)** In-Cell Western (ICW) assay of 14 and BIX-01294 in MDA-MB 231 cell lines, **(d)** Result of homogenous histone deacetylase assay of 14 alongside SAHA in K562 cell lines.

Most of the compounds retained G9a inhibition capabilities, indicated by the reduction in the ratio of the H3K9Me1 and H3K9Me2 peaks compared to the control reaction (Figure 6, see Supplementary Information Table 1). While fractionally less potent than parent BIX-01294, retention of inhibitory capabilities was nonetheless verification of the initial hypothesis. These result corresponded to the MALDI-TOF study done by a previously reported procedure [46]. With the knowledge that G9a activity preserved in the biochemical assay, next, we investigated the effect in the cell. H3K9Me2 cell immunofluorescence In-Cell Western (ICW) assay was used to assess G9a inhibition potential, and homogeneous cellular histone deacetylase assay used for measuring HDAC inhibition.

#### Functional potency evaluation for G9a inhibition

For assessing the functional potency of the dual inhibitors, we evaluated all the compounds by H3K9Me2 cell immunofluorescence In-Cell Western (ICW) assays and the results shown in Table 2. The MDA-MB-231 cell line was used in this study as this cell line possesses robust H3K9Me2 levels [5]. Our results suggested that compounds belonging to the class IV (southwest directing HDAC) exert a G9a activity comparable to the parent compound BIX-01294, but all other classes were significantly less potent.

**Table 2:** H3K9Me2 cell immunofluorescence In-Cell Western (ICW) assay results (MDA-MB-231 cell line)

Compound	G9a IC_50_ (μM)	Compound	G9a IC_50_ (μM)
4	96.69±1.68	4a	74.21±1.94
5	>100	5a	66.63±3.98
6	>100	6a	>100
7	76.74±0.89	7a	54.55±3.05
13	37.79±2.80	13a	>100
14	7.136±1.62	14a	46.83±1.97
15	72.10±1.37	15a	46.97±3.33
16	90.26±3.75	16a	45.71±1.76
19	99.63±3.13	21	ND
20	97.51±2.78	22	60.65±3.66
BIX-01294	4.563±1.2	5b	87.39±5.44

#### Functional potency evaluation for HDAC inhibition

The enzymatic activity of HDAC was measured in intact cells using the homogeneous cellular assay method [24]. A cell-permeable peptide Boc-K(Ac)-AMC used as the HDAC substrate, after deacetylation it is cleaved by trypsin to release the fluorescent 7-amino-4-methylcoumarin (AMC) and further quantified. Each compound candidate tested in both Hela and K562 cell lines, two compounds (13 and 14) showed significant HDAC inhibition (Table 3, see Supplementary Information Table 2 for full results). An evaluation of these structures indicated that only class III compounds displayed the desired activity, it was inconclusive if the R1 and R2 substitutions were responsible. Compound **5b**, synthesized from **3b** with a benzyl group at R1 and a methoxy at R2, did not show decent inhibition of HDAC compared with Compound **14**. Which indicated two important strategies for designing HDAC inhibitors with quinazoline core; an aromatic ring at R2 is essential for HDAC activity while the methoxy group at C6 of quinazoline core almost destroys HDAC inhibition. Linker lengths vary from Compound **11** to **14**, in which, compounds with 5 or 6 methylene groups between the lipophilic core and Zn binding domain found to be the best choice. Similarly, compounds **19-22** with the R1 substituted with the HDAC chain did not show promising activity, leaving **13** and **14** as the candidates for further study. Closer examination of the tested compounds indicated that HDAC activity is limited to compounds with a benzyl group at the 4-aminopiperidin ring (R1) along with no substitution at C6.

**Table 3:** Results of homogeneous cellular histone deacetylase assay

Compound	Hela IC_50_ (μM)	K562 IC_50_ (μM)
13	15.33±0.79	27.75±0.59
14	13.80±1.22	5.735±1.23
SAHA	5.044±0.53	2.056±0.59

### Molecular docking analysis

Molecular Docking analysis and molecular dynamics simulations were widely used to estimate the interactions between protein and small molecular compounds theoretically [47, 48]. Our assays found that **14** had good cellular potency for inhibition of both G9a and HDAC, so docking studies were used to examine the interactions of **14** with the target proteins compared to known ligands using Schrödinger Suite 2014-3 [49]. The crystal structure of human HDAC8 with MS-344 (PDB ID: 1T67) and human G9a with BIX-01294 (PDB ID: 3FPD) were employed as the templates for molecular docking studies [46, 50]. Initially we chose HDAC8 protein structure (PDB ID:1T69) for the docking study because it has SAHA (which we used as the control in cell based assays) as the co-crystallized ligand, but our study revealed a lower GLIDE score and docking score than the expected (Supplementary Information Table 3), so SP Glide algorithm was first validated by docking MS-344 and BIX-01294 from the complex back to the protein, ligand preparation was done using LigPrep with OPLS_2005. The search space was defined using Receptor Grid Generation in Glide, with the centroid of the ligand chosen to define the grid box. Standard precision mode was selected for validation docking, and default settings for scaling van der Waals radii were used. No constraints were defined for the docking runs. The docking pose with the highest score returned for MS-344 and BIX-01294 were compared with the starting protein complex. For subsequent molecular docking of compound **14** in the binding site of HDAC8 and G9a, LigPrep was used for energy minimizations of the molecule with the OPLS_2005 force field. Using the initial grids generated for HDAC8 and G9a, the standard precision docking was repeated for compound **14** as described above.

**Figure 7:**
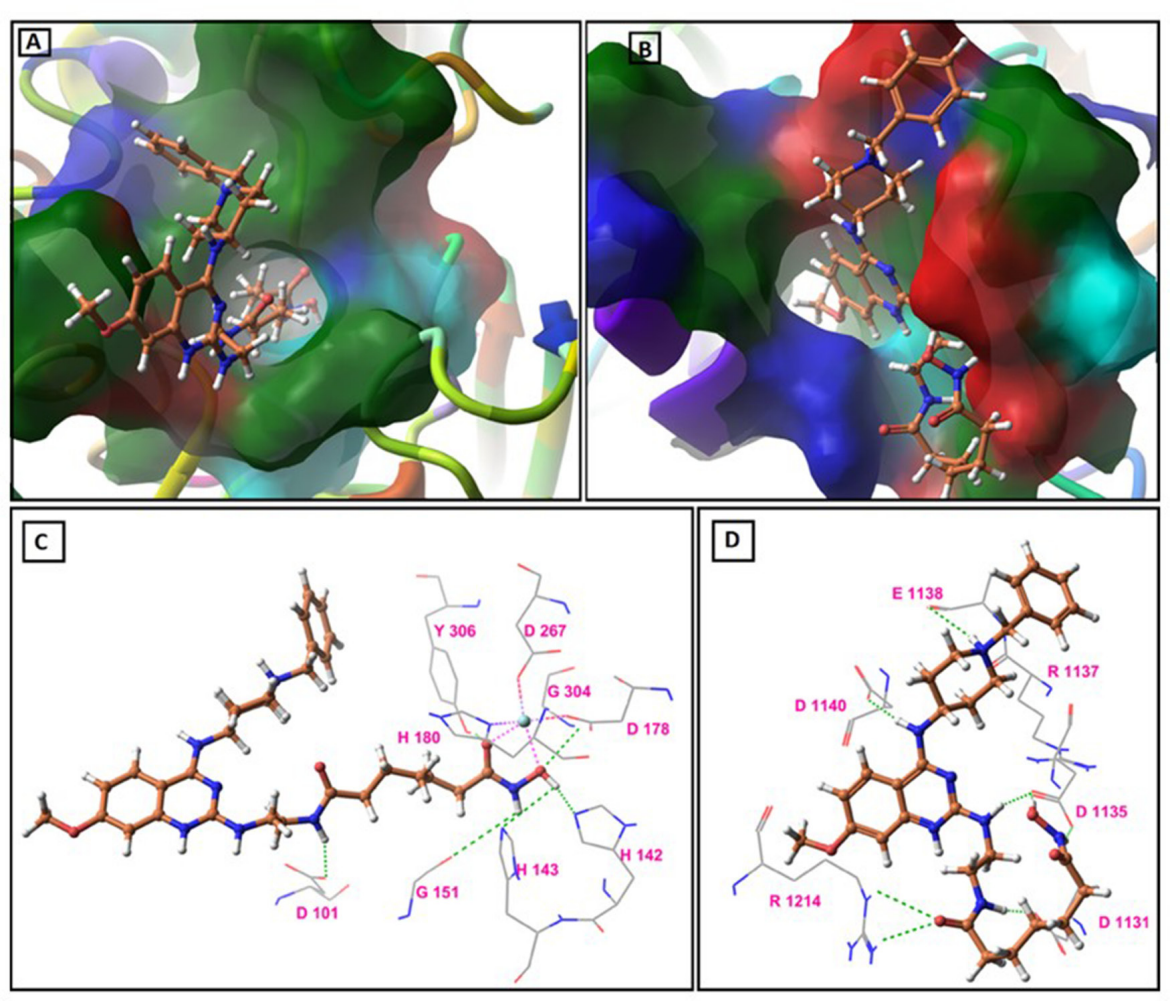
Molecular docking study results **(A)** Predicted binding mode of compound 14 on HDAC8 (PDB ID: 1T67), **(B)** Predicted binding mode of compound 14 on G9a (PDB ID: 3FPD), **(C)** Binding model of active compound 14 (orange) as revealed from GLIDE docking in the HDAC8 catalytic site (PDB ID: 1T67). The green dashed lines represent hydrogen bonds. The turquoise sphere represents Zn cation and with a trigonal bipyramidal coordination geometry. The pink dashed lines indicates the two contacts between the ligand and Zn cation, with the mixed dashed lines representing interaction between Zn cation and amino acid residues. H-bond distances (Å) between heteroatoms of ligand and amino acid residues are as follows: Asp101 (1.90), His142 (2.02), His143 (3.64), Gly151 (3.68), Gly304 (3.00), Tyr306 (2.17), **(D)** Binding model of active compound 14 (orange) as revealed from GLIDE docking in the G9a catalytic site (PDB ID: 3FPD). The green dashed lines represent hydrogen bonds. H-bond distances (Å) between heteroatoms of ligand and amino acid residues are as follows: Asp1131 (1.66), Asp1135 (1.75, 1.81), Arg1137 (3.33), Glu1138 (3.98), Asp1140 (1.77), Arg1214 (2.68, 2.90).

Table 4 and 5 showed the results of docking along with the prominent interactions for compound **14** with HDAC8 and G9a. Figure 7 illustrated the predicted binding modes and the detailed protein−inhibitor interactions of HDAC8 and G9a with compound **14**, respectively. Figure 7A showed that the catalytic tunnel of HDAC8 was occupied by the aliphatic side chain of the inhibitor, while the hydroxamate group chelated the zinc ion and hydrogen bonds with residues in the catalytic tunnel. The zinc ion displayed a trigonal bipyramidal geometry and two points contacted with the ligand (Figure 7A and 7C). Docking studies suggested important structural/catalytic roles for Gly140, His142, Gly151 and Gly304 in the active site and extending to Tyr306, Asp101. Moreover, compound **14** occupies the binding pocket in a similar posture as MS-344 in the catalytic site of HDAC8 (Supplementary Information Supplementary Figure 3).

**Table 4:** GLIDE docking results for MS-344 and compound 14 at the catalytic site of HDAC8 (PDB ID:1T67)

S. No.	Ligand ID	Docking Score	GLIDE score	Interactions		
				Hydrogen Bonds		Interaction with Zn^2+^
				Backbone	Side Chain	
1	MS-344	−7.931	−7.931	His142,His143,Gly151,Gly304;	Asp101, Tyr306;	Ionic interaction
2	**14**	−7.934	−8.369	Gly140,His142,Gly151,Gly304;	Asp101, Tyr306;	Ionic interaction

**Table 5:** GLIDE docking results for BIX-01294 and compound 14 at the catalytic site of G9a (PDB ID: 3FPD)

S. No.	Ligand ID	Docking Score	GLIDE score	Interactions		
				Hydrogen Bonds		Interaction with Zn^2+^
				Backbone	Side Chain	
1	BIX-01294	−7.664	−8.134	Ala1134	Asp1131,Asp1135,Asp1140;	NA
2	**14**	−7.321	−7.52	Arg1137, Glu1138	Asp1131,Asp1135,Asp1140,Arg1214;	NA

A similar study was performed to establish the binding characteristics of compound **14** with G9a. The binding model of compound **14** showed that it shares common hydrogen bonding interactions with key residues of the catalytic domain in a mode comparable to BIX-01294 (Supplementary Information Supplementary Figure 4). Most notably, the piperidine ring substituted at quinazolin-4-amine in compound **14** has hydrogen bonding interactions with Arg1137, Glu1138 residues, and the aliphatic chain was involved in some more hydrogen bonding interaction with the side chains of residues Asp1131, Asp1135, Asp1140 and Arg1214 (Figure 7B and 7D).

#### Cell anti-proliferation assay

Cell anti-proliferation assays were performed to determine the toxicity of these inhibitors. Several cell lines (MDA-MB-231, MCF-7 and A549) were incubated and then treated with varying concentrations of the inhibitors for 72 h, respectively. After the first cell culture screening, it was determined that the inhibitors were more effective with breast cell lines (MDA-MB-231 and MCF-7) compared to other cell lines, particularly compound **13** and **14** (Table 6, and Supplementary Information Table 4). These compounds were further evaluated against the control cell line HEK293 to test their toxicity with a non-cancerous cell line. As seen in Table 6, both SAHA and BIX-01294 appear to be toxic to cancer and normal cells, but compounds **13** and **14** displayed lower toxicity, particularly compound **14**. **14** also showed improved anti-proliferation abilities in all cancer cell lines and reduced toxicity in normal cell line compared to 13.

**Table 6:** Inhibition of compounds 13 and 14 on the growth of cancer cells and normal cells

compound	EC_50_ (μM)			
	MDA-MB-231^b^	MCF-7^c^	A549^d^	HEK293^e^
13	89.33±1.23	79.43±2.72	>100^a^	56.96±1.12
14	10.02±1.66	37.36±2.20	36.24±1.76	19.95±0.19
SAHA	2.874±0.84	8.124±4.98	19.31±1.26	2.482±1.13
BIX-01294	2.155±0.88	8.103±1.99	21.74±2.73	2.048±0.98

^a^>100 in the cases where the IC_50_ did not reach at the highest tested concentration (100 μM).

^b^MDA-MB-231: breast cancer cell line; ^c^MCF-7: breast cancer cell line; ^d^A549: human lung cancer cell line; ^e^HEK293 normal cell line; SAHA and BIX-01294 were used as the positive controls; Cells were exposed to the different inhibitors with various concentrations for 72h. Inhibition of cell growth by the listed compounds was determined by using CCK-8 kit. Data shown as mean ± SD of triplicates.

### ADME prediction studies

The same procedures and principals from the earlier *in silico* physico-chemical evaluations of known HDACIs were applied here to evaluate these novel dual inhibitors [42, 51]. ADMET module of Discovery Studio 3.1 was used to predict physical properties. Using Lipinski's rule of five [52], the octanol–water partition coefficient (AlogP98) should be less than 5. As seen in Table 7, the candidate compound **14** is well within accordance of the rule. In addition, other values also fell into the acceptable ranges of PSA-2D (7–200) and QplogS (−6.5 to 0.5), indicating **14** may possess good bioavailability. These parameters were also taken into consideration in identifying better inhibitors, suggesting that **14** has the characteristics desirable for a drug candidate.

**Table 7:** ADME prediction results

Entry	M.W	QPlogS^c^	PSA	PSA-2D^b^	AlogP98^a^
14	515.654	−3.702	161.25	141.462	2.511
SAHA	264.324	−2.139	102.256	81.037	1.838
BIX-01294	476.62	−6.792	50.675	63.249	4.189

^a^AlogP98 means atom-based LogP (octanol/water), ^b^PSA-2D means 2D fast polar surface area.

^c^QplogS means predicted aqueous solubility.

### Conclusions

Considering the inherent deficiency of HDACIs as a monotherapy, in conjunction with the past success of incorporating the HDAC pharmacophore into many dual inhibitors, we hypothesized that the core metal ion binding hydrophilic segment could be coupled with the lipophilic core of G9a inhibitors to increase the effectiveness. Both G9a and HDACs are therapeutic targets for cancer therapy and are both capable of targeting identical substrates (H3K9 and lysine 373 of p53). In search of a lead molecule featuring both HDAC and G9a inhibition, the H3 mimicking quinazoline core of G9a inhibitors used as a base scaffold. Next, several modifications at different sites introduced to cover most of the possible chemical space related to the position and chain length (linker gap between the metal binding portion and G9a core). From this design, we synthesized more than 20 compounds and tested biochemically and *in vitro* to see if they displayed the desired dual activity. Our primary assessment of success was from MALDI-TOF evaluation of the H3K9 methylation profile, many of the compounds retained G9a inhibition potential. Cell-based assays for all the compounds against several cell lines were used to determine their inhibition potential, and we found that **13** and **14** displays the desired dual activities comparable to the controls SAHA and BIX-01294. Cell toxicity of these compounds was determined using CCK-8, showing that compound **14** was both more effective and less toxic compared to **13**. The ADMET module of Discovery Studio 3.1 also predicted that the compound **14** has excellent physico-chemical properties, making it a viable drug candidate. Discovery of these small molecules with dual activity towards two epigenetic targets, HDACs and G9a, will provide the route for developing similar compounds with high potency soon. It is also worth mentioning that compound **14** may also act as a valuable tool in investigating the multi-targeting strategy, and its possible impacts on epigenetic targets. As this is the first time this sort of work has been done in respect to these two targets, it may also provide the basis to understanding histone cross-talk among distinct epigenetic targets. With this broad prospective, we further plan for a detailed SAR study specifically to compound **14** to provide a more potent dual inhibitor in conjunction with a mechanistic reasoning to understand the synergistic effect of inhibiting both HDACs and G9a methyltransferases.

### Experiment sections

Reagents were purchased from commercial suppliers Sigma-Aldrich, Alfa Aesar, TCI and Acros, and were used without further purification unless otherwise indicated. Anhydrous solvents (e.g., DMF, DIPEA, MeOH, DCM) were purchased from Sigma-Aldrich. The synthetic progress was monitored using silica gel 60 F254 thin layer chromatography plates (Merck EMD Millipore). Microwave reactions were performed using Initiator for organic synthesis. Column chromatography purifications were performed on an Isolera one system using SNAP columns with KP-Sil silica or Zip Si columns with KP-Sil normal phase silica cartridges (unless otherwise stated). The nuclear magnetic resonance spectra were recorded on a 400 MHz spectrometer by Topspin 3.1 with solvents of CDCl_3_and CD_3_OD. Chemical shifts described in ppm. Coupling constants, when reported, are described in hertz (Hz). High-resolution mass spectra (HRMS) data were acquired using orbitrap elite mass spectrometer with an electrospray ionization (ESI) source. All the samples were run under FT control at 600000 resolution. All temperatures are described in°C. The purity of all final compounds was confirmed by RP-HPLC analysis, was >95% or mentioned in the synthetic procedure. Analytical high performance liquid chromatography (HPLC) was performed using a Waters Agilent 1260 infinity, column used was Agilent eclipse plus C18 3.5 μM reverse phase 150 mm×4.6 mm chromatography column. Samples were detected using a wavelength of 254 nm. All samples were analyzed using acetonitrile (0.1% TFA): water (0.1% TFA) 5-60% over 30 min and a flow rate of 0.4 ml/min. Preparative HPLC was performed using the XBridge prep C18, 5 μM, 10×150 mm column and a flow rate of 1 ml/min.

### Synthesis

#### H_3_ (1-20, ARTKQTARKSTGGKAPRKQL)

Peptide was synthesized through Fmoc-Strategy. Automated peptide synthesis was performed on Liberty Blue Peptide Synthesizer. Peptide were synthesized under microwave-assisted protocols on Wang resins. The deblock mixture was 20% piperidine in DMF. The following Fmoc-Lys(Boc)-Wang resin from Nova biochem were employed. The Fmoc-protected amino acids were purchased from Chempep. Cocktail of TFA/TIS/Dodt/H_2_O (92.5:2.5:2.5:2.5) was used to cleave peptides off theresin. After cleavage, crude peptide was purified through a reverse phase C18 column (purchased from Agilent, Eclipse XDB-C18, 5 μm, 9.4*250mm).

#### Procedure A

General procedure for compounds **2, 2a and 2b**. 4-amino-piperidines (18.01 mmol) were added to a solution of 2,4-dichloro-6,7-dimethoxyquinazoline (2.11 g, 8.14 mmol in DMF 20 ml), followed by the addition of N,N-diisopropylethylamine (1.5 ml, 8.62 mmol) and the resulting mixture was stirred at room temperature for 2 h until TLC showed that the starting material had disappeared. Water was added to the reaction mixture, and the resulting solution was extracted with ethyl acetate. The organic layer was washed with 0.5% acetic acid aqueous solution and brine, dried and concentrated to give the crude product, which was purified on flash column via eluting with hexane-ethyl acetate (20%) to get 3.0g of the desired compound, yield 80-86%. Spectral properties of the product was matched with the reported compounds.

#### Procedure B

General procedure for compounds **3, 3a and 3b**. Compound **2** (6.0 mmol) was dissolved in 8 ml of isopropanol. To this solution was added tert-butyl (2-aminoethyl) carbamate (1.92 g, 12 mmol) and DIPEA (1.5 ml, 7.2 mmol). The resulting solution was placed inside a microwave at 160°C for 10 min. After cooling, TLC indicated the reaction was completed. Solvent was removed under reduced pressure, the residue was dissolved in DCM, washed with the saturated NaHCO_3_ solution. The combined organic phase was dried over Na_2_SO_4_ and concentrated under reduced pressure. The residue was purified on silica gel column, eluting with 5% MeOH in DCM (containing 0.5% Et_3_N) to give 1.8 g of the Boc–protected amino compound as pale yellow solid, yield 60-66%.

#### N2-(2-aminoethyl)-6,7-dimethoxy-N4-(1-methylpiperidin-4-yl)quinazoline-2,4-diamine (3)

Brown solid, 1.8 g, 66% yield. M.P. 109-107°C, ^1^H NMR (400 MHz, CDCl_3_) δ 7.10 (s, 1H), 7.06 (s, 1H), 6.19 (s, 1H), 4.33 – 4.20 (m, 1H), 3.91 (s, 6H), 3.17 (d, *J* = 5.4 Hz, 2H), 2.93 – 2.75 (m, 4H), 2.50 (s, 2H), 2.30 (s, 3H), 2.14 (m, 4H), 1.78 – 1.60 (m, 2H), 1.42 (s, 9H). ^13^C NMR (100 MHz, CDCl_3_) δ 159.5, 155.9, 154.8, 153.2, 148.9, 147.9, 107.1, 106.7, 101.3, 80.2, 56.6, 56.1, 54.5, 46.8, 45.0, 39.2, 30.4, 27.8. HRMS (ESI): *m/z*calcd for C_23_H_36_N_6_O_4_ [M + H]^+^, 461.2876; found, 461.2862.

#### N2-(2-aminoethyl)-6,7-dimethoxy-N4-(1-isopropylpiperidin-4-yl)quinazoline-2,4-diamine (3a)

Brown solid, 1.7 g, 60% yield. M.P. 114-116°C, ^1^H NMR (400 MHz, CDCl_3_) δ 7.23 (s, 1H), 7.19 (s, 1H), 4.13 (s, 1H), 3.84 (s, 6H), 3.58 – 3.44 (m, 4H), 3.39 (d, *J* = 4.9 Hz, 2H), 3.32 (d, *J* = 4.6 Hz, 2H), 2.96 – 2.83 (m, 2H), 2.11 (t, *J* = 11.3 Hz, 2H), 2.01 (d, *J* = 10.8 Hz, 2H), 1.74 – 1.59 (m, 2H), 1.40 (s, 9H), 1.01 (s, 6H).^13^C NMR (100 MHz, CDCl_3_) δ 165.1, 158.8, 156.3, 154.5, 145.6, 112.04, 108.9, 99.6, 80.8, 56.3, 55.9, 52.6, 49.9, 48.4, 41.4, 40.8, 31.7, 28.4. HRMS (ESI): *m/z*calcd for C_25_H_40_N_6_O_4_ [M + H]^+^, 489.3145; found, 489.3156.

#### N2-(2-aminoethyl)-6,7-dimethoxy-N4-(1-benzylylpiperidin-4-yl)quinazoline-2,4-diamine (3b)

Brown solid, 2.1 g, 64% yield. M.P. 130-132°C, ^1^H NMR (400 MHz, CDCl_3_) δ 7.36 – 7.14 (m, 5H), 6.79 (s, 1H), 6.58 (s, 1H), 6.01 (s, 2H), 5.44 (s, 1H), 4.11 (d, *J* = 12.4 Hz, 1H), 3.84 (m, 6H), 3.52 (s, 2H), 3.40 – 3.37 (m, 2H), 3.32 (d, *J* = 4.6 Hz, 2H), 2.93 – 2.84 (m, 2H), 2.11 (t, *J* = 11.3 Hz, 2H), 2.01 (d, *J* = 10.8 Hz, 2H), 1.75 – 1.59 (m, 2H), 1.38 (s, 9H).^13^C NMR (100 MHz, CDCl_3_) δ 165.1, 158.8, 156.3, 154.5, 145.6, 138.1, 129.2, 128.2, 127.1, 112.01, 108.9, 102.3, 78.9, 63.0, 56.3, 55.9, 52.6, 49.9, 48.4, 41.4, 40.8, 31.7, 28.3. HRMS (ESI): *m/z*calcd for C_29_H_40_N_6_O_4_ [M + H]^+^, 537.3189; found, 537.3165.

NH-Boc protection was removed to get the free amines of **3, 3a and 3b** using TFA/DCM overnight, dried amine was used directly in next step without further purification.

#### Procedure C

General procedure for compounds **4-7** and **4a-7a**, to a stirred solution of corresponding monomethyl ester (0.25 mmol) in anhydrous CH_2_Cl_2_ (5 ml) was added EDCI (70 mg, 0.35 mmol) followed by HOBt (50 mg, 0.35 mmol) at 0°C. After 30 min, a solution of compound **3** (138 mg, 0.3 mmol) and DIEPA (0.1 ml, 0.5 mmol) in CH_2_Cl_2_ (2 ml) was added drop-wise at 0°C. The mixture was allowed to stir at room temperature and monitored by TLC. Upon completion, the organic layer was washed with saturated aqueous NaHCO_3_ solution followed by brine. The organic extracts were dried over Na_2_SO_4_, filtered, and concentrated under reduced pressure. The crude product was purified by flash chromatography (MeOH/DCM up to 20%) to afford desired compounds as colorless oily liquid. HRMS (ESI): *m/z*calcd for C_27_H_42_N_6_O_5_ [M + H]^+^, 531.3295; found, 531.3279. This intermediate in methanol (2.5 ml) was added a solution of hydroxylamine (1 ml, 50% in water). The resulting solution was stirred for 3 h at 60°C. Then solvent was removed under vacuum and the crude residue purified by flash chromatography using reverse phase silica gel column using H_2_O (0.1% HCOOH)/CH_3_CN (0.1% HCOOH) as eluent (0-100 %). This afforded the expected derivatives as a yellow/brown sticky mass, 30-38% over 2 steps.

#### N1-(2-((6,7-dimethoxy-4-((1-methylpiperidin-4-yl) amino) quinazolin-2-yl) amino) ethyl)-N8-hydroxyoctanediamide (4)

44 mg, 33% yield. ^1^H NMR (400 MHz, MeOD) δ 7.81 (s, 1H), 7.59 (s, 1H), 6.91 (s, 1H), 4.68 (s, 1H), 3.93 (s, 6H), 3.64-3.47 (m, 5H), 3.23 (d, *J* = 1.4 Hz, 4H), 3.14 (m, 1H), 2.90 (d, *J* = 16.0 Hz, 3H), 2.36-2.28 (m, 2H), 2.21-2.07 (m, 5H), 1.64 – 1.49 (m, 4H), 1.30 (s, 4H). ^13^C NMR (100 MHz, MeOD) δ 183.7, 167.5, 148.4, 128.3, 124.8, 124.0, 117.1, 110.9, 104.0, 55.4, 54.7, 53.3, 52.8, 46.1, 42.1, 41.8, 38.2, 35.9, 32.3, 28.2, 25.4. HRMS (ESI): *m/z*calcd for C_26_H_41_N_7_O_5_ [M + H]^+^, 532.3247; found, 532.3248. HPLC purity 95.45% ; t_*R*_= 14.004.

#### N1-hydroxy-N8-(2-((4-((1-isopropylpiperidin-4-yl)amino)-6,7-dimethoxyquinazolin-2-yl)amino)ethyl)octanediamide (4a)

49 mg, 35% yield.^1^H NMR (400 MHz, MeOD) δ 7.76 (s, 1H), 7.70 (s, 1H), 6.90 (s, 1H), 4.72 (s, 1H), 3.92 (s, 6H), 3.60 (m, 5H), 3.48 - 3.38 (m, 4H), 3.33 (dd, *J* = 3.2, 1.6 Hz, 1H), 2.39-2.19 (m, 5H), 2.07 (t, *J* = 7.1 Hz, 2H), 1.56 (d, *J* = 5.5 Hz, 4H), 1.41-1.28 (m, 9H). ^13^C NMR (100 MHz, MeOD) δ 171.6, 166.3, 156.7, 153.1, 147.4, 147.2, 142.2, 135.7, 125.2, 124.7, 120.0, 117.3, 110.5, 104.1, 103.7, 57.9, 55.5, 45.0, 34.1, 32.1, 28.3, 25.0, 15.8. HRMS (ESI): *m/z*calcd for C_28_H_45_N_7_O_5_ [M + H]^+^, 560.3560; found, 560.3554. HPLC purity 95.12% ; t_*R*_= 14.820.

#### N1-(2-((6,7-dimethoxy-4-((1-methylpiperidin-4-yl)amino)quinazolin-2-yl)amino)ethyl)-N7-hydroxyheptanediamide (5)

38 mg, 30% yield. ^1^H NMR (400 MHz, MeOD) δ 7.70 (s, 1H), 6.97 (s, 1H), 4.69 (s, 2H), 3.96 (s, 6H), 3.85 (s, 1H), 3.67 – 3.49 (m, 4H), 3.47 (d, *J* = 5.7 Hz, 2H), 3.25 (d, *J* = 13.5 Hz, 2H), 3.15 (d, *J* = 7.4 Hz, 1H), 3.05 – 2.83 (m, 4H), 2.31 (d, *J* = 11.1 Hz, 2H), 2.23 – 2.06 (m, 5H), 1.64 – 1.52 (m, 3H), 1.35 (d, *J* = 6.9 Hz, 2H). ^13^C NMR (100 MHz, MeOD) δ 174.8, 170.0, 163.8, 156.6, 156.0, 153.4, 147.3, 112.0, 108.9, 99.6, 56.8, 53.6, 46.0, 40.5, 38.7, 37.4, 34.4, 30.4, 28.3, 25.2. HRMS (ESI): *m/z*calcd for C_25_H_39_N_7_O_5_ [M + H]^+^, 518.3091; found, 518.3080. HPLC purity 95.21% ; t_*R*_= 14.402.

#### N1-hydroxy-N7-(2-((4-((1-isopropylpiperidin-4-yl)amino)-6,7-dimethoxyquinazolin-2-yl)amino)ethyl)heptanediamide (5a)

51 mg, 38% yield. ^1^H NMR (400 MHz, MeOD) δ 7.75 (s, 1H), 7.61 (s, 1H), 6.94 (s, 1H), 4.73 (s, 1H), 3.94 (s, 6H), 3.61 (m, 5H), 3.48 (s, 2H), 3.42 – 3.23 (m, 3H), 2.39-2.21 (s, 5H), 2.09 (t, *J* = 6.9 Hz, 3H), 1.60 (s, 4H), 1.38 (m, 8H). ^13^C NMR (100 MHz, MeOD) δ 170.8, 167.7, 164.5, 155.5, 154.2, 152.0, 147.3, 110.8, 104.2, 98.1, 55.4, 53.4, 52.6, 48.2, 47.1, 38.5, 37.1, 35.4, 31.9, 28.5, 25.6, 15.8. HRMS (ESI): *m/z*calcd for C_27_H_43_N_7_O_5_ [M + H]^+^, 546.3405; found, 546.3385. HPLC purity 93.80% ; t_*R*_= 14.991.

#### N1-(2-((4-((1-benzylpiperidin-4-yl)amino)-6,7-dimethoxyquinazolin-2-yl)amino)ethyl)-N7-hydroxyheptanediamide (5b)

55 mg, 37% yield.^1^H NMR (400 MHz, MeOD) δ 7.67 (d, *J* = 1.9 Hz, 1H), 7.44 (dt, *J* = 15.7, 7.9 Hz, 5H), 6.96 (s, 1H), 4.48 (s, 1H), 3.96 (dd, *J* = 12.2, 3.3 Hz, 7H), 3.63 (s, 3H), 3.54 – 3.44 (m, 2H), 3.28 (d, *J* = 11.8 Hz, 2H), 2.72 (s, 2H), 2.32 – 2.16 (m, 5H), 2.10 (t, *J* = 7.1 Hz, 1H), 1.94 (d, *J*= 13.8 Hz, 2H), 1.60 (ddd, *J* = 15.4, 12.7, 7.5 Hz, 4H), 1.41 – 1.20 (m, 2H). 13C NMR (100 MHz, MeOD) δ 174.9, 171.4, 167.7, 159.4, 156.3, 156.0, 153.3, 147.4, 136.1, 130.6, 130.4, 129.1, 128.7, 128.6, 103.6, 98.4, 97.9, 60.5, 55.5, 51.1, 40.1, 38.3, 35.3, 28.5, 27.9, 25.0, 24.8. HRMS (ESI): *m/z*calcd for C_31_H_43_N_7_O_5_ [M + H]^+^, 594.3440; found, 594.3460. HPLC purity 96.20% ; t_*R*_= 14.001.

#### N1-(2-((6,7-dimethoxy-4-((1-methylpiperidin-4-yl)amino)quinazolin-2-yl)amino)ethyl)-N6-hydroxyadipamide (6)

44 mg, 35% yield. ^1^H NMR (400 MHz, MeOD) δ 7.65 (s, 1H), 6.93 (s, 1H), 4.69 (s, 1H), 3.95 (s, 6H), 3.60 (d, *J* = 14.8 Hz, 4H), 3.21 (m, 3H), 2.89 (d, *J* = 9.2 Hz, 4H), 2.29 (m, 4H), 2.13 (s, 3H), 1.90 (dt, *J* = 13.6, 6.7 Hz, 1H), 1.64 (s, 4H), 1.39 (d, *J* = 6.6 Hz, 2H). ^13^C NMR (100 MHz, MeOD) δ 170.8, 168.2, 159.3, 156.5, 156.2, 153.3, 147.3, 111.2, 108.6, 103.0, 55.5, 52.9, 46.0, 41.8, 40.56, 38.2, 37.4, 31.8, 28.2, 24.6. HRMS (ESI): *m/z*calcd for C_24_H_37_N_7_O_5_ [M + H]^+^, 504.2934; found, 504.2911. HPLC purity 96.81% ; t_*R*_= 13.374.

#### N1-hydroxy-N6-(2-((4-((1-isopropylpiperidin-4-yl)amino)-6,7-dimethoxyquinazolin-2-yl)amino)ethyl)adipamide (6a)

46 mg, 35% yield. ^1^H NMR (400 MHz, MeOD) δ 7.77 (s, 1H), 7.64 (s, 1H), 7.32 (s, 1H), 6.96 (s, 1H), 4.73 (s, 1H), 3.96 (s, 6H), 3.72 – 3.52 (m, 5H), 3.47 (s, 2H), 3.37 (d, *J* = 15.2 Hz, 2H), 2.39 (d, *J* = 12.1 Hz, 2H), 2.17 (m, 6H), 1.63 (s, 4H), 1.43 (d, *J* = 6.3 Hz, 6H). ^13^C NMR (100 MHz, MeOD) δ 174.5, 170.5, 168.0, 157.1, 156.0, 153.5, 147.3, 113.4, 108.4, 89.7, 57.2, 55.8, 49.2, 49.5, 40.8, 39.7, 38.0, 32.5, 28.2, 24.7, 15.5. HRMS (ESI): *m/z*calcd for C_26_H_41_N_7_O_5_ [M + H]^+^, 532.3247; found, 532.3245. HPLC purity 96.95% ; t_*R*_= 14.164.

#### N1-(2-((6,7-dimethoxy-4-((1-methylpiperidin-4-yl)amino)quinazolin-2-yl)amino)ethyl)-N5-hydroxyglutaramide (7)

40 mg, 33% yield. ^1^H NMR (400 MHz, MeOD) δ 7.68 (s, 1H), 7.63 (s, 1H), 7.30 (s, 1H), 6.84 (s, 1H), 4.66 (s, 1H), 3.92 (s, 6H), 3.61 (s, 4H), 3.46 (s, 2H), 3.35 (d, *J* = 15.4 Hz, 3H), 2.90 (m, 3H), 2.29 (m, 4H), 2.14 (d, *J* = 6.6 Hz, 4H), 1.91 (s, 2H). ^13^C NMR (100 MHz, MeOD) δ 170.8, 168.2, 159.4, 159.3, 156.5, 153.3, 147.3, 136.1, 122.1, 111.2, 108.6, 103.7, 55.5, 52.9, 46.1, 41.8, 40.0, 38.4, 38.2, 35.2, 31.8, 28.2, 24.6. HRMS (ESI): *m/z*calcd for C_23_H_35_N_7_O_5_ [M + H]^+^, 490.2778; found, 490.2756. HPLC purity 95.61% ; t_*R*_= 12.751.

#### N1-hydroxy-N5-(2-((4-((1-isopropylpiperidin-4-yl)amino)-6,7-dimethoxyquinazolin-2-yl)amino)ethyl)glutaramide (7a)

50 mg, 38% yield.^1^H NMR (400 MHz, MeOD) δ 7.67 (s, 1H), 6.93 (s, 1H), 4.72 (s, 2H), 3.94 (s, 6H), 3.59 (d, *J* = 12.4 Hz, 5H), 3.47 (s, 2H), 3.36 (d, *J* = 13.9 Hz, 2H), 2.37 (s, 2H), 2.22-2.12 (m, 6H), 1.63 (m, 4H), 1.44 (s, 6H). ^13^C NMR (100 MHz, MeOD) δ 173.1, 168.0, 158.5, 156.5, 153.3, 134.9, 147.5, 110.6, 103.7, 98.5, 57.8, 55.2, 40.1, 38.4, 35.7, 32.4, 31.7, 28.5, 16.2, 15.8. HRMS (ESI): *m/z*calcd for C_25_H_39_N_7_O_5_ [M + H]^+^, 518.3091; found, 518.3139. HPLC purity 93.38% ; t_*R*_= 13.670.

### Compounds 13-16 and 13a-16a

#### 2,4-dichloro-7-methoxyquinazoline

Compound 10 was prepared according to the previously reported procedure [45], 3.4 g of anthranilic acid (20 mmol) and 3.5 equiv. of urea were finely powdered using mortar and pestle and heated to 200°C in a round-bottom flask open to the atmosphere. After 2 h, the mixture was cooled, triturated with water, and filtered to give the product as crude. Product was dried and used in next step directly. Molecular ion peak for C_9_H_8_N_2_O_3_ was found at 192.0773. Crude quinazoline-2,4-dione and 2.4 g of N,N-diethylaniline were mixed in 45 ml of phosphorus oxychloride, and the mixture was refluxed overnight under an argon atmosphere. The crude reaction mixture was concentrated, neutralized the excess of POCl_3_ using NaHCO_3_ and extracted to EA, dried on Na_2_SO_4_ and evaporated, purified using flash column, eluting at 20% of EA/Hexane. White fluffy powder, 1.82 g, 40% overall yield. HRMS (ESI): *m/z*calcd for C_9_H_6_Cl_2_N_2_ [M + H]^+^, 228.9935; found, 228.9934.

#### N-(1-benzylpiperidin-4-yl)-2-chloro-7-methoxyquinazolin-4-amine (11) and 2-chloro-N-(1-isopropylpiperidin-4-yl)-7-methoxyquinazolin-4-amine (11a)

Compound **11** and **11a** were prepared according to the procedure A, using 1-methylpiperidin-4-amine or 1-benzylpiperidin-4-amine. **11**: Yellow powder, 74%. M.P. 134-136°C, ^1^H NMR (CDCl_3_, 400 MHz) δ ppm 7.54 (d, J = 9.0 Hz, 1H), 7.27-7.34(m, 5H), 7.10 (d, J = 2.4 Hz, 1H), 7.04 (dd, J1 = 9.0 Hz, J2 = 2.4 Hz, 1H), 5.61(d, J = 7.71 Hz, 1H), 4.23-4.33 (m, 1H), 3.88 (s, 3H), 3.57 (s, 2H), 2.91 (d, J = 11.9 Hz, 2H), 2.24-2.30 (m, 2H), 2.08-2.13 (m, 2H), 1.59-1.69 (m, 2H). ^13^C NMR (CDCl_3_, 100 MHz) δ ppm 163.7, 129.8, 158.3, 153.3, 137.7, 129.3, 128.3, 127.3, 122.0, 117.9, 107.2, 106.9, 62.9, 55.7, 52.0, 48.0, 31.9. HRMS (ESI): *m/z*calcd for C_21_H_23_ClN_4_O [M + H]^+^, 383.1639; found, 383.1610.

**11a**: Brownish yellow semi solid, 86%.^1^H NMR (400 MHz, CDCl_3_) δ 7.56 (d, *J* = 9.1 Hz, 1H), 7.13 (s, 1H), 7.07 (d, *J* = 9.1 Hz, 1H), 4.28 (s, 1H), 3.92 (s, 3H), 2.88 (d, *J* = 11.0 Hz, 2H), 2.35 (s, 3H), 2.26 (t, *J* = 11.6 Hz, 2H), 2.17 (d, *J* = 12.2 Hz, 2H), 1.86 (s, 1H), 1.66 (m, 2H). HRMS (ESI): *m/z*calcd for C_15_H_19_ClN_4_O [M + H]^+^, 306.1247; found, 307.1323.

#### N2-(2-aminoethyl)-N4-(1-benzylpiperidin-4-yl)-7-methoxyquinazoline-2,4-diamine (12) and N2-(2-aminoethyl)-7-methoxy-N4-(1-methylpiperidin-4-yl)quinazoline-2,4-diamine (12a)

Compounds 12 and 12a were obtained via **Procedure B**:

**12**: Brown solid, 1.94 g, 64%. ^1^H NMR (400 MHz, CDCl_3_) δ 8.75 (s, 2H), 8.49 (d, *J* = 5.2 Hz, 1H), 7.96 (s, 1H), 7.37 – 7.14 (m, 4H), 6.62 (d, *J* = 8.9 Hz, 2H), 5.56 (s, 1H), 4.22 (s, 1H), 4.06 – 3.88 (m, 1H), 3.67 (d, *J* = 10.9 Hz, 3H), 3.51 (d, *J* = 9.7 Hz, 4H), 3.26 (s, 2H), 2.99 (dd, *J* = 14.9, 7.4 Hz, 1H), 2.90 (s, 2H), 2.09 (d, *J* = 15.0 Hz, 4H), 1.91 (d, *J* = 12.4 Hz, 3H), 1.28 (d, *J* = 10.5 Hz, 10H). HRMS (ESI): *m/z*calcd for C_28_H_38_N_6_O_3_ [M + H]^+^, 507.3084; found, 507.3047.

**12a**: Brown solid, 1.75 g, 68%. ^1^H NMR (400 MHz, CDCl_3_) δ 7.46 (d, *J* = 8.9 Hz, 1H), 6.84 (s, 1H), 6.74 (d, *J* = 8.8 Hz, 1H), 5.55 (s, 2H), 4.18 (s, 1H), 3.88 (s, 3H), 3.61 (d, *J* = 3.9 Hz, 2H), 3.39 (d, *J* = 4.8 Hz, 2H), 2.87 (d, *J* = 11.2 Hz, 2H), 2.34 (s, 3H), 2.21 (t, *J* = 11.3 Hz, 2H), 2.12 (d, *J* = 11.8 Hz, 2H), 1.66 (d, 10.4 Hz, 2H), 1.44 (s, 9H). HRMS (ESI): *m/z*calcd for C_22_H_34_N_6_O_3_ [M + H]^+^, 431.2771; found, 431.2767.

Compounds **13-17** and **13a-17a** were synthesized according to procedure C from the corresponding free amines, Yield varied from 30-40%, yellow/brown sticky solids were obtained after purification.

#### N1-(2-((4-((1-benzylpiperidin-4-yl)amino)-7-methoxyquinazolin-2-yl)amino)ethyl)-N8-hydroxyoctanediamide (13)

45 mg, 31% yield.^1^H NMR (400 MHz, MeOD) δ 8.06 (s, 1H), 7.52 (d, *J* = 13.6 Hz, 5H), 6.83 (d, *J* = 11.2 Hz, 2H), 4.62 (s, 1H), 4.34 (s, 2H), 3.89 (s, 3H), 3.70 – 3.40 (m, 6H), 3.22 – 3.03 (m, 3H), 2.21 (m, 8H), 1.56 (s, 4H), 1.29 (s, 5H). ^13^C NMR (100 MHz, MeOD) δ 175.0, 171.5, 165.1, 159.8, 154.0, 151.3, 141.9, 130.9, 129.7, 129.6, 128.8, 125.4, 113.7, 102.9, 98.0, 59.9, 55.3, 50.7, 40.0, 38.3, 35.7, 32.2, 28.4, 28.3, 27.9, 25.4, 25.1. HRMS (ESI): *m/z*calcd for C_31_H_43_N_7_O_4_ [M + H]^+^, 578.3455; found, 578.3444. HPLC purity 95.41%; t_*R*_= 16.756.

#### N1-hydroxy-N7-(2-((7-methoxy-4-((1-methylpiperidin-4-yl)amino)quinazolin-2-yl)amino)ethyl)heptanediamide (13a)

44 mg, 33% yield. ^1^H NMR (400 MHz, CDCl_3_) δ 7.46 (s, 1H), 6.85 – 6.49 (m, 2H), 4.20 (s, 1H), 3.87 (s, 3H), 3.70 – 3.46 (m, 4H), 2.95 (d, *J* = 10.6 Hz, 2H), 2.36 (s, 3H), 2.23 (dd, *J* = 21.2, 12.2 Hz, 6H), 2.15 – 2.00 (m, 6H), 1.81 (d, *J* = 10.6 Hz, 3H), 1.65 (s, 5H). ^13^C NMR (100 MHz, MeOD) δ 175.0, 168.6, 165.2, 153.8, 149.2, 134.8, 125.4, 113.7, 108.7, 102.9, 98.0, 55.2, 52.8, 46.2, 42.2, 40.0, 38.2, 35.6, 32.2, 28.4, 28.3, 28.1, 25.3, 25.0. HRMS (ESI): *m/z*calcd for C_25_H_39_N_7_O_4_ [M + H]^+^, 502.3142; found, 502.3143. HPLC purity 95.75%; t_*R*_= 13.767.

#### N1-(2-((4-((1-benzylpiperidin-4-yl)amino)-7-methoxyquinazolin-2-yl)amino)ethyl)-N7-hydroxyheptanediamide (14)

49 mg, 35% yield. ^1^H NMR (400 MHz, MeOD) δ 8.10 (d, *J* = 9.1 Hz, 1H), 7.59 – 7.40 (m, 5H), 6.97 (d, *J* = 9.1 Hz, 1H), 6.90 (s, 1H), 4.60 (s, 1H), 4.18 (s, 2H), 3.94 (s, 3H), 3.64 (d, *J* = 8.0 Hz, 2H), 3.46 (dd, *J* = 14.1, 8.1 Hz, 4H), 3.39 – 3.31 (m, 2H), 3.18 – 2.96 (m, 2H), 2.23 (dd, *J* = 19.2, 11.7 Hz, 4H), 2.15 – 1.92 (m, 4H), 1.69 – 1.53 (m, 4H), 1.41 – 1.27 (m, 2H). ^13^C NMR (100 MHz, MeOD) δ 175.0, 171.4, 167.8, 165.3, 159.9, 154.0, 141.9, 131.2, 130.5, 129.1, 128.7, 125.3, 113.8, 103.0, 98.2, 60.5, 55.1, 51.0, 40.2, 38.2, 35.3, 32.0, 28.4, 28.0, 25.0, 24.8. HRMS (ESI): *m/z*calcd for C_30_H_41_N_7_O_4_ [M + H]^+^, 564.3298; found, 564.3307. HPLC purity 95.02%; t_*R*_= 16.600.

#### N1-hydroxy-N7-(2-((7-methoxy-4-((1-methylpiperidin-4-yl)amino)quinazolin-2yl)amino)ethyl) heptanediamide (14a)

49 mg, 40% yield. ^1^H NMR (400 MHz, MeOD) δ 8.10 (s, 1H), 7.17 (s, 1H), 6.94 (m, 1H), 4.62 (d, *J* = 12.4 Hz, 2H), 3.94 (s, 3H), 3.86 (s, 2H), 3.73 – 3.55 (m, 2H), 3.47 (s, 1H), 3.29 (m, 5H), 3.15 (dd, *J* = 14.9, 7.5 Hz, 2H), 2.97 – 2.77 (m, 4H), 2.45 – 2.17 (m, 3H), 2.24 – 2.02 (m, 3H), 1.61 (s, 2H), 1.42 – 1.22 (m, 2H). ^13^C NMR (100 MHz, CDCl_3_) δ 178.5, 174.4, 166.0, 165.6, 159.2, 153.1, 129.78, 117.7, 115.8, 106.9, 59.6, 53.5, 50.7, 46.8, 42.1, 39.7, 39.3, 36.0, 32.0, 29.8, 28.4. HRMS (ESI): *m/z*calcd for C_24_H_37_N_7_O_4_ [M + H]^+^, 487.2907; found, 488.2962. HPLC purity 94.16%; t_*R*_= 13.232.

#### N1-(2-((4-((1-benzylpiperidin-4-yl)amino)-7-methoxyquinazolin-2-yl)amino)ethyl)-N6-hydroxyadipamide (15)

52 mg, 38% yield. ^1^H NMR (400 MHz, MeOD) δ 8.03 (s, 1H), 7.52 (m, 6H), 6.78 (d, *J* = 10.3 Hz, 2H), 4.61 (s, 1H), 4.36 (s, 2H), 3.87 (s, 3H), 3.60 (m, 4H), 3.46 (m, 2H), 3.38 (m, 3H), 2.49 – 1.78 (m, 8H), 1.64 (s, 4H). ^13^C NMR (100 MHz, MeOD) δ 171.25, 167.79, 165.02, 159.78, 153.88, 139.89, 131.05, 129.70, 129.50, 128.93, 113.70, 111.3, 103.5, 66.8, 59.84, 55.36, 50.73, 41.6, 39.2, 38.4, 35.38, 32.02, 27.80. HRMS (ESI): *m/z*calcd for C_24_H_37_N_7_O_4_ [M + H]^+^, 550.3142; found, 550.3148. HPLC purity 96.48%; t_*R*_= 16.262.

#### N1-hydroxy-N6-(2-((7-methoxy-4-((1-methylpiperidin-4-yl)amino)quinazolin-2-yl)amino)ethyl)adipamide (15a)

41 mg, 35% yield.^1^H NMR (400 MHz, MeOD) δ 8.08 (s, 1H), 6.87 (d, *J* = 13.0 Hz, 2H), 4.68 (s, 2H), 3.88 (d, *J* = 12.3 Hz, 3H), 3.82 – 3.69 (m, 1H), 3.62 (s, 4H), 3.46 (s, 2H), 3.22 (m, 2H), 3.28 – 3.17 (m, 1H), 3.19 – 3.01 (m, 1H), 2.89 (d, *J* = 15.2 Hz, 3H), 2.42 – 2.19 (m, 3H), 2.09 (d, *J* = 29.5 Hz, 2H), 1.90 (s, 1H), 1.62 (s, 3H), 1.38 (d, *J* = 6.5 Hz, 2H). ^13^C NMR (100 MHz, MeOD) δ 173.7, 170.0, 167.9, 164.5, 159.2, 153.4, 141.4, 124.6, 113.0, 102.3, 97.4, 54.4, 53.6, 52.2, 46.2, 45.5, 41.6, 39.3, 37.5, 34.3, 34.0, 27.5. HRMS (ESI): *m/z*calcd for C_23_H_35_N_7_O_4_ [M + H]^+^, 474.2829; found, 474.2807. HPLC purity 96.40%; t_*R*_= 12.879.

#### N1-(2-((4-((1-benzylpiperidin-4-yl)amino)-7-methoxyquinazolin-2-yl)amino)ethyl)-N5-hydroxyglutaramide (16)

52 mg, 39% yield.^1^H NMR (400 MHz, MeOD) δ 8.01 (s, 1H), 7.51 (d, *J* = 18.6 Hz, 5H), 6.76 (d, *J* = 17.4 Hz, 2H), 4.49 (d, *J* = 13.1 Hz, 3H), 3.86 (s, 3H), 3.53 (d, *J* = 9.5 Hz, 5H), 3.22 (s, 3H), 2.23 (m, 6H), 1.93 (s, 4H). ^13^C NMR (100 MHz, MeOD) δ 174.1, 170.8, 167.9, 165.0, 159.7, 153.6, 141.7, 131.0, 129.0, 129.8, 128.3, 125.4, 115.4, 113.6, 102.8, 67.9, 59.9, 55.3, 50.7, 40.1, 38.3, 34.8, 31.7, 27.8, 21.6. HRMS (ESI): *m/z*calcd for C_23_H_35_N_7_O_4_ [M + H]^+^, 536.2985; found, 536.2998. HPLC purity 94.34% ; t_*R*_= 16.051.

#### N1-hydroxy-N5-(2-((7-methoxy-4-((1-methylpiperidin-4-yl)amino)quinazolin-2-yl)amino)ethyl)glutaramide (16a)

35 mg, 31% yield. ^1^H NMR (400 MHz, MeOD) δ 8.10 (s, 1H), 6.87 (d, *J* = 11.2 Hz, 2H), 4.70 (s, 1H), 3.92 (s, 3H), 3.73 (dd, *J* = 12.9, 6.5 Hz, 1H), 3.66 (m, 3H), 3.46 (s, 2H), 3.35 (d, *J* = 15.5 Hz, 2H), 3.23 (m, 1H), 2.88 (d, *J* = 14.5 Hz, 2H), 2.46 – 2.21 (m, 4H), 2.15 (s, 2H), 1.90 (s, 2H), 1.37 (m, 4H).^13^C NMR (100 MHz,) δ 175.8, 171.2, 166.0, 165.2, 153.3, 151.3, 127.6, 113.2, 110.1, 103.0, 57.8, 54.6, 50.5, 46.9, 41.8, 39.2, 36.4, 33.6, 31.4, 19.0. HRMS (ESI): *m/z*calcd for C_22_H_33_N_7_O_4_ [M + H]^+^, 460.2672; found, 460.2648. HPLC purity 95.90%; t_*R*_= 12.615.

#### N-(1-benzylpiperidin-4-yl)-6,7-dimethoxy-2-(4-methyl-1,4-diazepan-1-yl)quinazolin-4-amine (18)

Compound **18** was synthesized according to the previously reported procedure [44], which was treated with Pd/C under H_2_ gas to get the free amine. HRMS (ESI): *m/z*calcd for C_21_H_32_N_6_O_2_ [M + H]^+^, 401.2264; found, 401.2642. This amine was directly used in procedure C while using monomethyl suberate ester to get **19** and monomethyl pimelate to obtain **20**.

#### 8-(4-((6,7-dimethoxy-2-(4-methyl-1,4-diazepan-1-yl)quinazolin-4-yl)amino)piperidin-1-yl)-N-hydroxy-8-oxooctanamide (19)

48 mg, 34% yield over 2 steps. ^1^H NMR (400 MHz, MeOD) δ 7.68 (s, 1H), 7.20 (s, 1H), 4.66 (d, *J* = 12.0 Hz, 1H), 4.51 (s, 1H), 4.22 (s, 2H), 4.12 (d, *J* = 12.7 Hz, 1H), 3.96 (m, 8H), 3.45 (s, 2H), 3.28 (d, *J* = 9.7 Hz, 3H), 2.83 (d, *J* = 7.7 Hz, 4H), 2.47 (dd, *J* = 15.0, 7.3 Hz, 2H), 2.36 (s, 2H), 2.16 (m, 4H), 1.65 (m, 6H), 1.40 (s, 5H). ^13^C NMR (100 MHz, MeOD) δ 172.6, 171.5, 167.5, 158.5, 155.8, 152.9, 147.6, 103.4, 102.7, 99.6, 56.3, 55.5, 55.4, 44.7, 43.7, 42.7, 40.7, 32.4, 32.2, 31.5, 30.6, 28.5, 28.3, 25.1, 25.0, 24.3. HRMS (ESI): *m/z*calcd for C_29_H_45_N_7_O_5_ [M + H]^+^, 572.3516; found, 572.3530. HPLC purity 94.71%; t_*R*_= 15.347.

#### 7-(4-((6,7-dimethoxy-2-(4-methyl-1,4-diazepan-1-yl)quinazolin-4-yl)amino)piperidin-1-yl)-N-hydroxy-7-oxoheptanamide (20)

62 mg, 45% yield over two steps. ^1^H NMR (400 MHz, MeOD) δ 7.72 (s, 1H), 7.23 (s, 1H), 4.66 (d, *J* = 12.1 Hz, 1H), 4.52 (s, 1H), 4.26 (s, 2H), 4.12 (d, *J* = 12.0 Hz, 2H), 3.96 (d, *J* = 14.8 Hz, 8H), 3.49 (d, *J* = 39.9 Hz, 4H), 2.86 (dd, *J* = 25.3, 14.7 Hz, 4H), 2.64 – 2.29 (m, 4H), 2.27 – 1.92 (m, 4H), 1.83 – 1.50 (m, 6H), 1.50 – 1.34 (m, 2H). ^13^C NMR (100 MHz, MeOD) δ 172.5, 171.4, 167.2, 158.5, 155.9, 152.5, 147.7, 137.0, 103.5, 102.7, 99.2, 56.18, 55.6, 55.5, 49.4, 45.9, 44.6, 43.5, 42.4, 40.2, 32.4, 32.1, 31.4, 30.6, 28.2, 25.0, 24.8, 24.0. HRMS (ESI): *m/z*calcd for C_28_H_43_N_7_O_5_ [M + H]^+^, 558.3404; found, 558.3387. HPLC purity 96.04%; t_*R*_= 14.311.

### Compounds 21 and 22

Compound **10** was treated with NHBoc ethyline diamine as the procedure A to get the intermediate **11b**, which was further treated with 1-methyl-1,4-diazepane in accordance to procedure B to yield **18a**, followed by procedure C, using monomethyl suberate or monomethyl pimelate to get **21** and **22**.

#### tert-butyl (2-((2-chloro-7-methoxyquinazolin-4-yl)amino)ethyl)carbamate (11b)

78% yield.^1^H NMR (400 MHz, CDCl_3_) δ 7.70 (d, *J* = 9.0 Hz, 2H), 7.08 – 6.88 (m, 2H), 5.39 (s, 1H), 3.85 (s, 3H), 3.67 (d, *J* = 3.9 Hz, 2H), 3.57 – 3.37 (m, 2H), 1.40 (s, 9H). HRMS (ESI): *m/z*calcd for C_16_H_21_ClN_4_O_3_+ [M + H]^+^, 353.1380; found, 353.1372.

#### tert-butyl(2-((7-methoxy-2-(4-methyl-1,4-diazepan-1-yl)quinazolin-4-yl)amino)ethyl) carbamate (18a)

69% yield.^1^H NMR (400 MHz, CDCl_3_) δ 7.56 (d, *J* = 8.9 Hz, 1H), 6.95 (s, 1H), 6.85 (s, 1H), 6.63 (s, 1H), 5.52 (s, 1H), 4.04 – 3.93 (m, 2H), 3.86 (s, 3H), 3.62 (d, *J* = 4.9 Hz, 2H), 3.44 (d, *J* = 4.5 Hz, 3H), 2.73 (s, 2H), 2.69 – 2.51 (m, 2H), 2.37 (s, 3H), 2.03 (s, 2H), 1.41 (s, 9H). HRMS (ESI): *m/z*calcd for C_22_H_34_N_6_O_3_[M + H]^+^, 431.2771; found, 431.2746.

#### N1-hydroxy-N8-(2-((7-methoxy-2-(4-methyl-1,4-diazepan-1-yl)quinazolin-4 yl)amino)ethyl)octanediamide (21)

36 mg, 29% yield over 2 steps.^1^H NMR (400 MHz, MeOD) δ 7.97 (d, *J* = 8.7 Hz, 1H), 7.16 (s, 1H), 7.02 (d, *J* = 8.6 Hz, 1H), 4.28 (s, 2H), 3.94 (s, 3H), 3.87 (s, 2H), 3.74 (d, *J* = 5.6 Hz, 3H), 3.60 – 3.48 (m, 4H), 3.42 (s, 2H), 2.89 (d, *J* = 7.3 Hz, 3H), 2.39 (s, 2H), 2.21 (t, *J* = 7.0 Hz, 4H), 1.55 (m, 4H), 1.29 (s, 4H). ^13^C NMR (100 MHz, MeOD) δ 175.5, 167.49 164.98 159.76 153.4, 124.9, 114.1, 103.6, 99.8, 55.3, 55.1, 48.2, 48.0, 47.8, 47.6, 47.4, 47.2, 46.9, 46.0, 43.5, 42.6, 41.3, 37.5, 35.6, 34.0, 28.5, 28.4, 25.4, 24.7, 24.0. HRMS (ESI): *m/z*calcd for C_25_H_39_N_7_O_4_[M + H]^+^, 502.3142; found, 502.3128. HPLC purity 96.21%; t_*R*_= 13.810.

#### N1-hydroxy-N7-(2-((7-methoxy-2-(4-methyl-1,4-diazepan-1-yl)quinazolin-4-yl)amino)ethyl)heptanediamide (22)

43 mg, 36% yield over 2 steps.^1^H NMR (400 MHz, MeOD) δ 7.98 (d, *J* = 9.1 Hz, 1H), 7.14 (d, *J* = 1.9 Hz, 1H), 7.00 (m, 1H), 4.27 (s, 2H), 3.93 (s, 5H), 3.88 (s, 1H), 3.88 – 3.66 (m, 4H), 3.52 (m, 5H), 3.42 (s, 2H), 2.90 (s, 3H), 2.39 (s, 1H), 2.24 (dd, *J* = 10.9, 7.1 Hz, 5H), 1.84 (dd, *J* = 13.7, 6.5 Hz, 2H). ^13^C NMR (100 MHz, MeOD) δ 174.99, 171.13, 168.17, 164.87, 159.67, 153.28, 125.03, 114.13, 103.56, 99.82, 55.19, 43.67, 42.61, 41.15, 37.62, 35.26, 31.88, 24.93, 24.60. HRMS (ESI): *m/z*calcd for C_24_H_37_N_7_O_4_[M + H]^+^, 488.2985; found, 488.2960. HPLC purity 96.80%; t_*R*_= 13.370.

**7-(benzyloxy)-N-(1-isopropylpiperidin-4-yl)-6-methoxy-2-(4-methyl-1,4-diazepan-1-yl)quinazolin-4-amine (24)** was synthesized from **1a**, by following procedure A (**23**) and procedure B (**24**), then benzyl group was removed using Pd catalyzed hydrogenolysis, mixture of compound **24** (600 mg, 1.2 mmol) and 10 wt% Pd(OH)_2_/C (90 mg) in ethanol (100 ml) was stirred for 40 h at room temperature under hydrogen balloon. The reaction mixture was filtered and concentrated to provide the debenzylated product **4-((1-isopropylpiperidin-4-yl)amino)-6-methoxy-2-(4-methyl-1,4-diazepan-1-yl) quinazolin-7-ol (25)** as brownish yellow solid, 90 %.

#### N-hydroxy-7-((4-((1-isopropylpiperidin-4-yl)amino)-6-methoxy-2-(4-methyl-1,4-diazepan-1-yl)quinazolin-7-yl)oxy)heptanamide (26)

**Procedure D**, Ethyl heptanoate (80 μl, 0.4 mmol) was added to the ice cold solution of compound **24** (200 mg, 0.4 mmol) in DMF and K_2_CO_3_ (280 mg, 2 mmol), reaction mixture was warmed to room temperature and then at 60°C. After 6 h reactions, mixture was evaporated to get the residue and dissolved in DCM and washed with brine, organic layer was vacuum dried and eluted in flash column using reverse phase silica at 40 % ACN/H_2_O to get the intermediate ester, which was then dissolved in 2 ml of MeOH and treated with 50% NH_2_OH/water mixture (1 ml) overnight to afford the targeted product. Reaction mixture was dried and purified using reverse column and further by HPLC using ACN (0.1% HCOOH) /H_2_O (0.1% HCOOH) as eluent. 94 mg, 40% overall yield. ^1^H NMR (400 MHz, MeOD) δ 7.61 (s, 1H), 7.06 (s, 1H), 4.14 (s, 3H), 3.97 (d, J = 14.7 Hz, 6H), 3.50 (s, 4H), 3.15 (s, 3H), 3.05 (s, 3H), 2.74 – 2.57 (m, 3H), 2.38 (s, 3H), 2.22 – 2.03 (m, 3H), 1.90 (d, J = 14.1 Hz, 3H), 1.63 (d, J = 8.4 Hz, 4H), 1.48-1.42 (m, 3H), 1.40-1.31 (m, 6H). ^13^C NMR (100 MHz, MeOD) δ 170.9, 164.2, 158.0, 155.5, 152.0, 146.6, 110.9, 108.5, 101.5, 70.3, 57.4, 56.8, 56.6, 53.1, 46.4, 44.3, 32.4, 29.8, 29.2, 28.8, 26.8, 25.1, 24.1, 20.3. HRMS (ESI): *m/z*calcd for C_30_H_49_N_7_O_4_ [M + H]^+^, 572.3924; found, 572.3925. HPLC purity 96.80%; t_*R*_= 13.370.

#### 8-((4-((1-benzylpiperidin-4-yl)amino)-2-(4-methyl-1,4-diazepan-1-yl)quinazolin-7-yl)oxy)- N-hydroxyoctanamide (30)

Targeted analog **30** was synthesized from the anthranillic acid starting material.

#### 7-(benzyloxy)-N-(1-benzylpiperidin-4-yl)-2-(4-methyl-1,4-diazepan-1-yl)quinazolin-4-amine (27)

prepared according to the procedure A and B. ^1^H NMR (400 MHz, CDCl_3_) δ 7.90 (d, *J* = 3.6 Hz, 1H), 7.26-7.18 (m, 5H), 7.12 (s, 1H), 6.98 (d, *J* = 3.9, 1H), 5.87 (s, 1H), 4.39-4.12 (m, 5H), 3.92 (s, 3H), 3.63 (s, 2H), 3.20 (d, *J* = 4.6 Hz, 2H), 3.04 (d, *J* = 2.4 Hz, 2H), 2.52 (d, *J* = 2.4 Hz, 2H), 2.28 (s, 3H), 1.82-1.66 (m, 6H). ^13^C NMR (100 MHz, CDCl_3_) δ 163.0, 159.0, 158.6, 138.4, 129.1, 128.2, 127.0, 122.2, 112.1, 104.8, 104.3, 63.1, 58.8, 57.2, 55.3, 52.4, 48.2, 46.6, 45.9, 45.8, 32.0, 27.6. MALDI-TOF: *m/z* for C_27_H_36_N_6_O [M + H]^+^ is 461.9.

#### 4-((1-benzylpiperidin-4-yl)amino)-2-(4-methyl-1,4-diazepan-1-yl)quinazolin-7-ol (28)

Aryl demethylation using BBr3 was employed [53]. BBr3 solution in DCM (1 ml, 1M) was added to the ice cold solution of compound **27** (450 mg, 1 mmol), resulting solution was allowed to be in normal room temperature and stirred the reaction mixture under inert atmosphere. Reaction was monitored using mass spec (MALDI-TOF), after completion of the reaction at about 48 h, water was added to the mixture and basified with NaHCO_3_, extracted with DCM, washed with brine and dried to obtain a pale yellow solid and used for next steps without purification. MALDI-TOF: *m/z* for C_26_H_34_N_6_O [M + H]^+^ is 447.8, ratio of the product was over 90% to starting material.

#### 8-((4-((1-benzylpiperidin-4-yl)amino)-2-(4-methyl-1,4-diazepan-1-yl)quinazolin-7-yl)oxy)- N-hydroxyoctanamide (30)

Procedure D using **28** as the starting material, afforded the desired intermediate as colorless solid. HRMS (ESI): *m/z*calcd for C_35_H_50_N_6_O_3_[M + H]^+^, 603.4022; found, 603.4031. Subsequently, compound **29** was dissolved in 2 ml of MeOH and treated with 50% NH_2_OH/water mixture (1 ml) overnight to afford the targeted product. Reaction mixture was dried and purified using reverse column and further by HPLC using ACN/H_2_O as eluent. Fractions collected were concentrated and lyophilized to get brown powder, 54 mg, 23% yield over two steps. ^1^H NMR (400 MHz, MeOD) δ 8.10 (d, *J* = 9.2 Hz, 1H), 7.65 – 7.53 (m, 4H), 7.06 (d, *J* = 2.2 Hz, 1H), 6.94 (d, *J* = 8.9 Hz, 1H), 3.94 (d, *J* = 9.0 Hz, 2H), 3.80 (s, 2H), 3.72 – 3.59 (m, 4H), 3.50 (s, 2H), 3.22 (s, 3H), 2.48 (s, 2H), 2.34 (d, *J* = 14.2 Hz, 3H), 2.15 (s, 2H), 2.05 (s, 4H), 1.96 (s, 5H), 1.86 (s, 2H), 1.68 (s, 2H), 1.46 (s, 4H). ^13^C NMR (100 MHz, MeOD) δ 175.5, 167.4, 164.9, 159.7, 153.4, 140.3, 129.1, 128.2, 127.0, 124.9, 114.1, 109.0, 103.6, 68.7, 63.7, 55.3, 55.1, 46.0, 43.5, 42.6, 41.3, 34.0, 28.5, 28.4, 25.4, 24.7. HRMS (ESI): *m/z*calcd for C_33_H_47_N_7_O_3_ [M + H]^+^, 590.3819; found, 590.3832. HPLC purity 96.26%; t_*R*_= 14.192.

### Molecular docking analysis

#### Protein preparation and grid generation

The coordinates for the HDAC8/MS-344 complex (PDB ID: 1T67) and G9a/BIX-01294 complex (PDB ID: 3FPD) were downloaded from the RCSB Protein Data Bank. In these structures, MS-344 and BIX-01294 are bound to HDAC8, G9a respectively. The PDB protein-ligand structures were processed with the Protein Preparation Wizard in the Schrödinger suite. The protein structure integrity was checked and adjusted, and missing residues and loop segments near the active site were added using Prime. The receptor was prepared for docking by the addition of hydrogen atoms and the removal of co-crystallized molecules except for Zn^2+^, as it is near to the active site in the case HDAC. Active site water molecules outside 5.0 Å from the ligand were removed. The bound ligands were used to specify the active site. A 3D box was generated around each ligand to enclose the entire vicinity of active site. The receptor grid for each target was prepared with the help of OPLS_2005 force field. The grid center was set to be the centroid of the co-crystallized ligand, and the cubic grid had a size of 20 Å.

#### Ligand preparation

the 2D ligand structures were prepared using ChemBioDraw Ultra 12.0, and the 3D structures were generated by Schrödinger suite. Schrödinger's LigPrep program was used to generate different conformations of ligands. All possible protomers and ionization states were enumerated for **14** and bound ligands using Ionizer at a pH of 7.4. Tautomeric states were generated for chemical groups with possible prototropic tautomerism.

#### Molecular docking

molecular docking studies were performed by using a GLIDE docking module of Schrödinger suite. It performs grid-based ligand docking with energetics and searches for positive interactions between ligand molecules and a typically larger receptor molecule, usually a protein. Finally, prepared ligands were docked into the generated receptor grids using Glide SP docking precision. The results were analyzed on the basis of the GLIDE docking score and molecular recognition interactions. All the 3D figures were obtained using Schrödinger Suite 2014-3.

### Cloning, protein expression and purification

Mouse histone methyltransferase G9a (969-1263) cDNA was amplified from the cDNA of BALB/c mouse thymus, and the fragment was sub-cloned into a vector with a 6His-sumo tag. The mouse G9a (mG9a) was expressed in *Escherichia coli* BL21 (DE3) by the addition of 1 mM isopropyl-1-thio-D-galactopyranoside (IPTG) and incubated overnight at 16°C.

The 6His-sumo mG9a (969-1263) protein was purified using the following procedure: harvested cell pellet was re-suspended in 20 mM Tris (pH 8.0), 500 mM NaCl, 0.1% β-mercaptoethanol, and 1 mM PMSF. Cells were lysed by sonicating for 15 s with 6 s intervals for a total time of 15 min on an ice bath. The supernatant of cell lysate was loaded onto a Ni^+^ affinity column (Invitrogen) then washed with buffer (20 mM Tris-HCl pH 8.0, 500 mM NaCl, 20 mM imidazole, 0.1% β-mercaptoethanol, and 1 mM PMSF). The 6His-sumo tag was cleaved from the column by adding Ubiquitin-like-specific protease 1 (ULP-1) at 4°C for 12 h. Wash buffer was then run through the Ni^+^ column again and the elution buffer collected. Subsequently, advanced protein purification was done by HiTrap Q HP sequential Superdex 200 10/300 GL. Elute of every step was analyzed by SDS PAGE, stained by Coomassie brilliant blue (CBB).

## MALDI-TOF-MS

The *in vitro* inhibition of G9a by the synthesized compounds were measured by MALDI-TOF mass spectrum (Bruker MALDI TOF/TOF Analyzer). 400 nM purified G9a, 5 μM synthesized histone H3 (1-21) and 10 μM non-radioactive S-adenosyl methionine (Sigma) were added in reaction buffer (50 mM HEPES pH 8.0, 5 μg/ml BSA and 0.1% β-Mercaptoethanol) with or without inhibitors (5 μM). The reaction was incubated at room temperature for 30 min, and stopped by TFA. 1 μl of the sample was mixed with CHCA matrix and m/z peaks were obtained at reflection positive mode. The results of mass spectrum were analyzed using the Bruker flex analysis software, a detailed description of data processing is provided as supplementary information (S3).

### Cell based assays

#### Cell lines information

MDA-MB-231(breast cancer cell line), MCF-7 (breast cancer cell line), A549 (human lung cancer cell line), K562 (human immortalized myelogenous leukemia cell line), Hela (human cervical cancer cell line), HEK293 (normal cell line).

#### Reagents

CCK-8, Trichostatin A and trypsin were purchased from Sigma.

#### Cell line

MDA-MB-231, A549 cell lines were grown at 37°C/5% CO_2_ in Dulbecco's Modified Eagle's Medium(from Sigma) supplemented with 10% fetal bovine serum and 2% 200 mM L-glutamine and 0.5% antibiotic-antimycotic solution(from Sigma).

Hela, K562 cell lines were grown at 37°C/5% CO_2_ in RPMI 1640 medium (Gibco)supplemented with 10% fetal bovine serum and 0.5% antibiotic-antimycotic solution.

MCF-7 cell line was grown at 37°C/5% CO_2_ in Eagle's Minimum Essential Medium supplemented with 10% fetal bovine serum and 0.5% antibiotic-antimycotic solution.

### HDAC activity assay

The manual assay was developed by Thomas's group [54]. HeLa cells were seeded into white 96-well cell culture plates (corning costar 3596) at a density of 8000-10000 cells/well (total volume 81 μl culture medium) and incubated under standard cell culture conditions(37°C, 5% CO_2_). After 24 h, 9 μl inhibitors with different concentration were added to the HeLa cells and incubation was continued for 3 h under cell culture conditions. After this treatment period, 10 μl of a 2 mM stock solution of the substrate Boc-K(Ac)-AMC was added into the 96 well plates with Hela cells and inhibitors. Cell culture plates were incubated under standard cell culture conditions for an additional 3 h before addition of 100 μl/well lysis/developer buffer mix (50 mM Tris–HCl, pH 8.0, 137 mM NaCl, 2.7 mM KCl, 1 mM MgCl_2_, 1 vol% Nonidet-P40, 2.0 mg/ml trypsin, 10 μM TSA). After final incubation for 3 h under cell culture conditions, fluorescence was measured at excitation of λex = 355 nm and emission of λem = 460 nm on the Perkin–Elmer Wallac Victor V 1420 multilabel plate reader (Perkin–Elmer, Wellesley, USA). A549 and K562 cell lines used the same method, respectively. IC_50_s were calculated using GraphPad Prizm statistical package with sigmoidal variable slope dose response curve fit.

#### G9a H3K9me2 cellular assay

Cells were seeded at 8000-10000 cells (100 μl) in black-walled 96-well plates (Thermo 165305) and exposed to various inhibitor concentrations for 48 h. After the incubation, the media was removed and 100 μl fixation and permeabilization solution (2% formaldehyde in PBS) for fixation was added for 30 min. And then use 200 μl 0.1% Triton X100 in PBS washing solution to wash (allow wash to shake on a plate shaker for 5 min). After five washes, cells were blocked for 1 h with 150 μl blocking buffer to each well (1% BSA in PBS) (allow blocking at room temperature with moderate shaking on a plate shaker). After 1 h, remove the blocking buffer from the blocking step and add primary antibody in blocking buffer to cover the bottom of each well. (Three out of four replicates were exposed to the primary H3K9me2 antibody, Abcam no. 1220 at 1/500 dilution in 1% BSA, PBS for overnight, one replicate was reserved for the background control (only blocking buffer). The wells were washed five times with 0.1% Tween 20 in PBS, then secondary IR800 conjugated antibody (LiCor) and cell tag 700 stain added for 1 h. (incubate for 1 h with gentle shaking at room temperature, protect plate from light during incubation). After 5 washes with 0.1% Tween 20 in PBS, remove wash solution completely from wells. Turn the plate upside down and tap or blot gently on paper towels to remove traces of wash buffer. The plates were read on an Odyssey CL_X_ (LiCor) scanner at both 800 nm (H3K9me2 signal) and 700 nm (cell tag 700 stain signal) channels. IC_50_s were calculated using GraphPad Prizm statistical package with sigmoidal variable slope dose response curve fit.

#### Toxicity assay

A549, MDA-MB-231, MCF-7 and HEK293 cells were seeded at 8000-10000 cells (100 μl) in white 96-well plates and pre-incubate the plate for 24 h under standard cell culture conditions, respectively. And then the cells were exposed to the different inhibitors with various concentrations for 72 h. Finally, 10 μl of CCK-8 kit solution was added to each well and incubated for 3-4 hours under standard cell culture conditions, and the 96 well plates were measured the absorbance at 450 nm using Perkin–Elmer Wallac Victor ^3^V 1420 multi label plate reader (Perkin–Elmer, Wellesley, USA). EC_50_s were calculated using GraphPad Prizm statistical package with sigmoidal variable slope dose response curve fit.

## SUPPLEMENTARY MATERIALS FIGURES AND TABLES



## Abbreviations

EHMT2: (Euchromatic histone-lysine N-methyltransferase 2), lysine methyltransferases 1c(KMT1c)
GLP: (G9a like protein)
