# Supplementary material for: Structure based design, synthesis and activity studies of small hybrid molecules as HDAC and G9a dual inhibitors
Source: Oncotarget. 2017 Jun 28;8(38):63187–207. doi: 10.18632/oncotarget.18730 (PMC5609913; doi:10.18632/oncotarget.18730)

Structure based design, synthesis and activity studies of small hybrid molecules as HDAC and G9a dual inhibitors

SUPPLEMENTARY MATERIALS

Combination study of BIX-01294 and SAHA

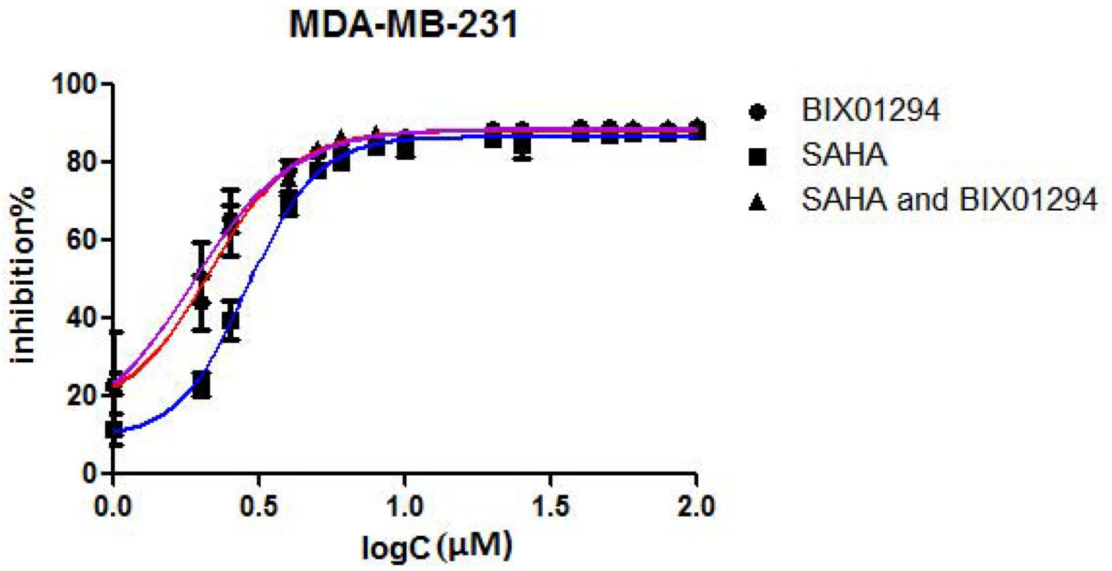

Supplementary Figure 1: EC_{50} plot of combination study, BIX01294 and SAHA (each 1-100 μM) were plotted alongside BIX01294 + SAHA (1-100 μM in a 1:1 ratio).

MALDI-TOF study of methylation

MALDI-TOF based experiment was performed according to the protocol developed by Chang etal.[1] MALDI spectrums were collected using Bruker flex control software and analyzed by flex analysis. After labelling each cluster peaks of H3K9Me0, H3K9Me1 and H3K9Me2 for all of the tested concentrations, area under the cluster (AUC) were extracted by using the same flex analysis software. % abundance of each peak was calculated by following formula,

A = % Abundance of (H3K9Me0) = area of H3K9Me0/ (area of H3K9Me0+area of H3K9Me1 + area of H3K9Me2)

B = % Abundance of (H3K9Me1) = area of H3K9Me1/ (area of H3K9Me0+area of H3K9Me1 + area of H3K9Me2)

C = % Abundance of (H3K9Me2) = area of H3K9Me2/ (area of H3K9Me0+area of H3K9Me1 + area of H3K9Me2).

This was repeated for each spectra (3 multiples for each samples).

G9a catalyze dimethylation of H3K9 and hence formation of H3K9Me2 was considered as the product formation and H3K9Me0 and H3K9Me1 is considered substrate not modified to the final product. Hence here % conversion to product is also C, from this to get the %maximal activity (%MA), C was compared to the % conversion when no inhibitor was used (D).

Finally % inhibition was found by subtracting $\%MA_{(i)}$ from 100

Example: % methylation levels and % inhibition when used 5 μM compound **14**

A = 13.09

B = 61.18

C = 25.72

% conversion when no inhibitor was used D = 84.43 (average of six measurements)

%MA = 100 * 25.72/84.43

= 30.46

Finally % inhibition = 100 – 30.46

= **69.54%**

Average of 3 values were reported in the Supplementary Figure 2 and Supplementary Table 1.

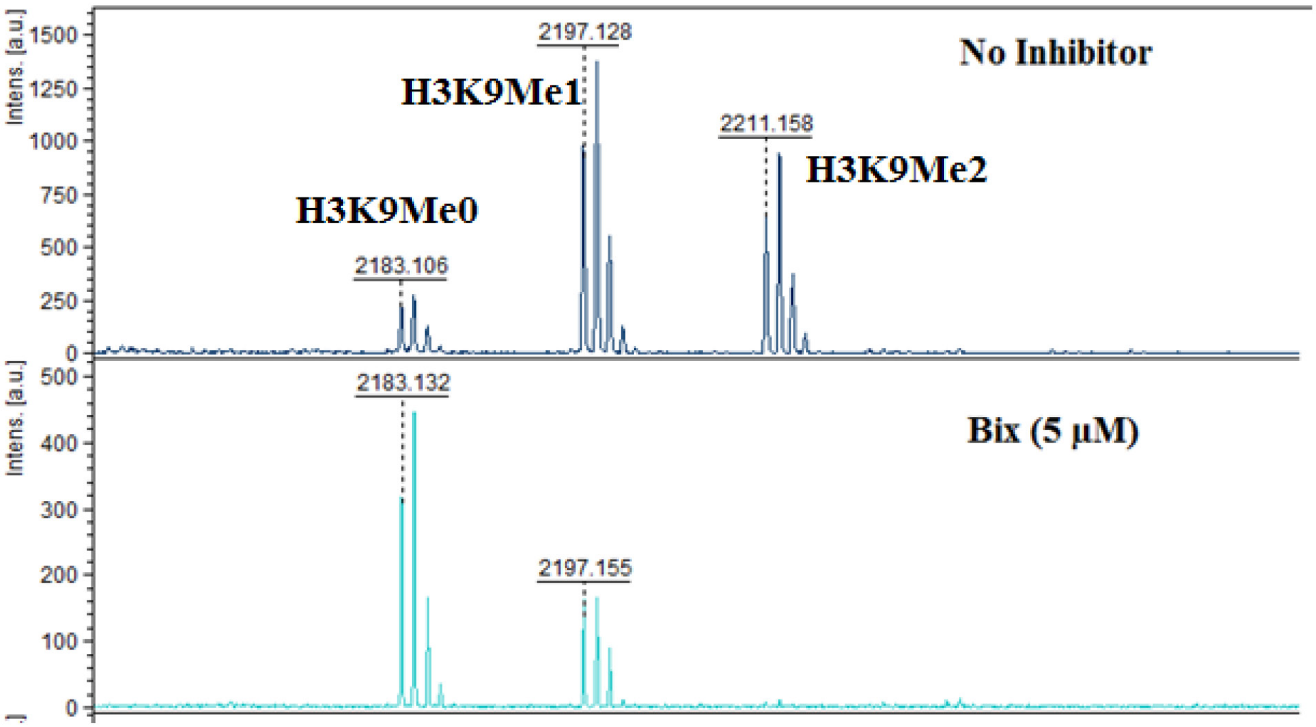

Supplementary Figure 2: A sample MALDI-TOF image used for the % inhibition analysis.

Supplementary Table 1: MALDI-TOF methylation study of inhibitors at 5 μM concentration for 30 min

CPD#	Inhibition (%)	CPD#	Inhibition (%)
**4**	26.96±12.15	**4a**	11.22±3.68
**5**	65.02±1.95	**5a**	47.55±9.32
**6**	26.64±11.34	**6a**	50.65±16.51
**7**	19.58±20.95	**7a**	15.01±15.73
**13**	24.91±17.42	**13a**	62.00±14.44
**14**	29.68±23.32	**14a**	69.55±3.43
**15**	69.12±24.54	**15a**	47.85±12.88
**16**	40.32±13.81	**16a**	40.32±13.81
**19**	10.71±25.88	**20**	14.62±15.81
**21**	22.75±26.72	**22**	40.26±15.33
**5b**	38.01±4.99	**BIX-01294**	77.68±5.73

HDAC assay results

Supplementary Table 2: Cell based homogenous HDAC assay results

CPD#	IC_{50}-HDAC		
	Hela[c]	A549[d]	K562[e]
4	NA[a]	NA	NA
4a	NA	NA	NA
5	NA	NA	NA
5a	NA	NA	NA
6	NA	NA	NA
6a	NA	NA	NA
7	NA	NA	NA
7a	NA	NA	NA
13	15.33±0.79	>100	27.75±0.59
13a	>100	>100	>100
14	13.80±1.22	>100	5.735±1.23
14a	>100[b]	>100	>100
15	>100	>100	>100
15a	>100	>100	>100
16	>100	>100	>100
16a	>100	>100	>100
19	>100	>100	>100
20	>100	>100	>100
21	>100	>100	>100
22	>100	>100	>100
BIX	NA	NA	NA
SAHA	5.044±0.53	>100	2.056±0.59

NA[a] not active up to the highest concentration tested (the highest concentration of all compounds is 100 µM;
>100[b] in the cases where the IC_{50} did not reach at the highest tested concentration (100 µM);
[c]Hela: human cervical cancer cell line; [d]A549: human lung cancer cell line; [e]K562: human immortalized myelogenous leukemia cell line; SAHA was used as the positive control. Data are shown as mean ± SD of triplicate.

Molecular docking study results

HDAC (PDB ID:1T69) protein interactions study

Initially we chose HDAC8 protein structure (PDB ID:1T69) for the docking study because it has SAHA (which we used as the control in cell based assays) as the co-crystallized ligand, but our study revealed a lower GLIDE score and docking score than the expected (Supplementary Table 3), and so we did a similar study on another HDAC8 protein structure 1T67 and found a higher binding scores and chose this for later study.

Supplementary Table 3: Glide docking study results for compound 14 and SAHA at the catalytic site of HDAC8 (PDB ID: 1T69)

S.NO.	LIGAND ID	GLIDE SCORE	DOCKING SCORE	INTERACTIONS			
				H- bonds		π-π	Interaction with Zn^{2+} atom
				Backbone	Side chain		
1	**SAHA**	-5.794	-5.794	His142, His143, Gly151, Gly304	Asp101, Tyr306	Phe152	+
2	**14**	-8.858	-8.471	Gly140, His142, Gly151, Gly206, Phe207, Pro209, Gly304	Asp101, Tyr306	-	+

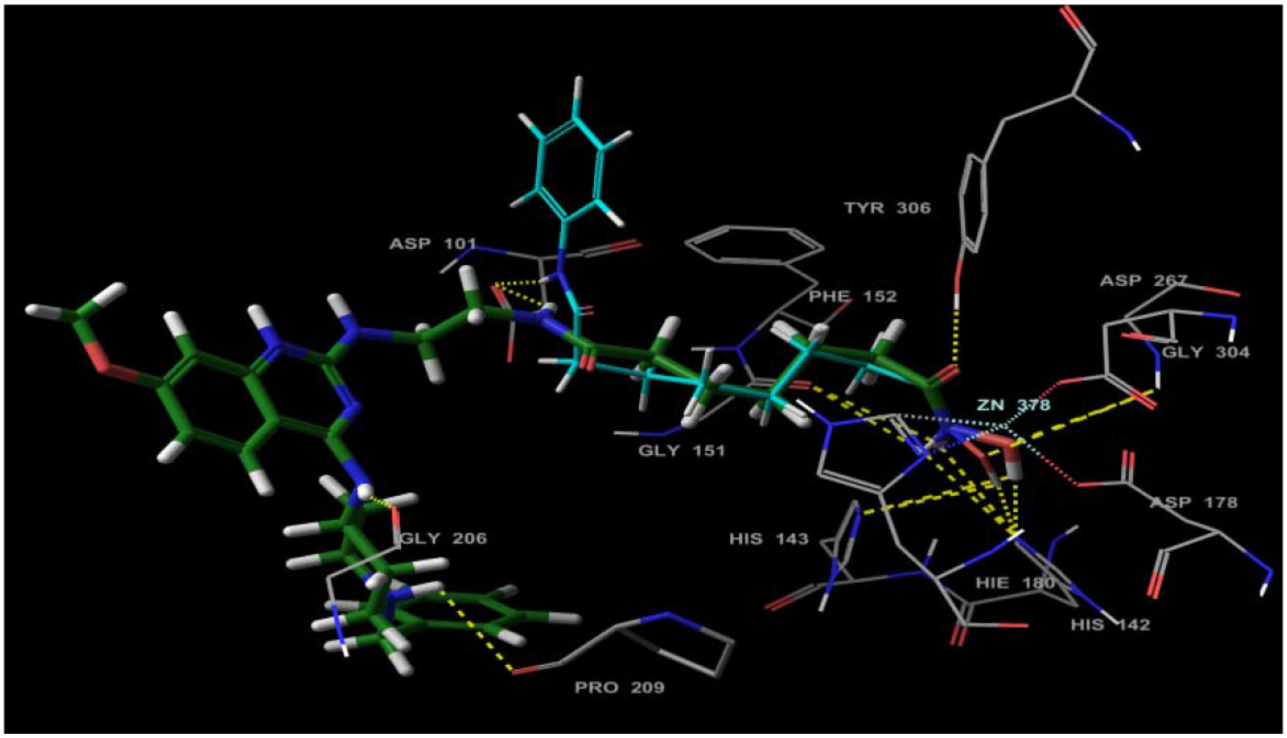

Supplementary Figure 3: Binding analysis image of compound 14 and SAHA with HDAC8 (PDB ID: 1T69).

Superimposed images of 14, MS-344 with HDAC8 (PDB ID: 1T67) and 14, BIX-01294 with G9a (PDB ID: 3FPD)

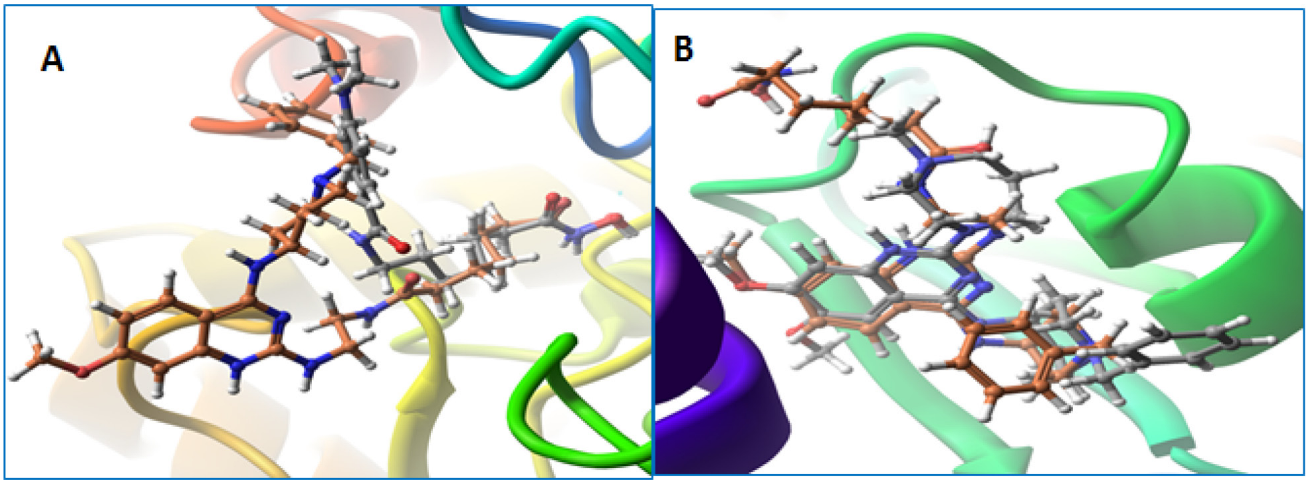

Supplementary Figure 4: Super imposed ligands with their corresponding target enzyme. **(A)** Superimposition of cocrystallized MS-344 (gray), best docked pose of **14** (orange) in the catalytic site of HDAC8 (PDB ID: 1T67), **(B)** Superimposition of BIX-01294 (gray), best docked pose of **14** (orange) in the catalytic site of G9a (PDB ID: 3FPD).

Cytotoxicity study results

Supplementary Table 4: Detailed results of cytotoxicity study

CPD#	EC_{50}(μM)		
	MDA-MB-231[c]	MCF-7[d]	A549[e]
4	>100[b]	>100	NA[a]
4a	>100	>100	NA
5	>100	>100	NA
5a	>100	>100	NA
6	>100	>100	NA
6a	>100	>100	NA
7	>100	>100	NA
7a	>100	>100	NA
13	89.33±1.23	79.43±2.72	>100
13a	>100	>100	NA
14	10.02±1.66	37.36±2.20	36.24±1.76
14a	82.32	>100	NA
15	95.15	>100	NA
15a	77.62	>100	NA
16	38.15	57.29	>100
16a	90.54	>100	NA
19	>100	>100	NA
20	>100	>100	NA
21	>100	>100	NA
22	>100	>100	NA
26	31.28±3.30	>100	NA
30	24.01±3.64	>100	NA
5b	12.29±3.27	74.57±1.81	NA
BIX-01294	2.155±0.88	8.103±1.99	21.74±2.73
SAHA	2.874±0.84	8.124±4.98	19.31±1.26

NA[a], not active up to the highest concentration tested (the highest concentration of all compounds is 100 μM.
>100[b] in the cases where the IC_{50} did not reach at the highest tested concentration (100 μM).
[c]MDA-MB-231: breast cancer cell line; [d]MCF-7: breast cancer cell line; [e]A549: human lung cancer cell line; SAHA and BIX-01294 are used as the positive controls;
Cells were exposed to the different inhibitors with various concentrations for 72 h, Inhibition of cell growth by the listed compounds was determined by using CCK-8 kit. Data are shown as mean ± SD of triplicate.

NMR, HPLC spectra of compounds (14, 5b, 20, 21, 30)

HPLC of compound **14**

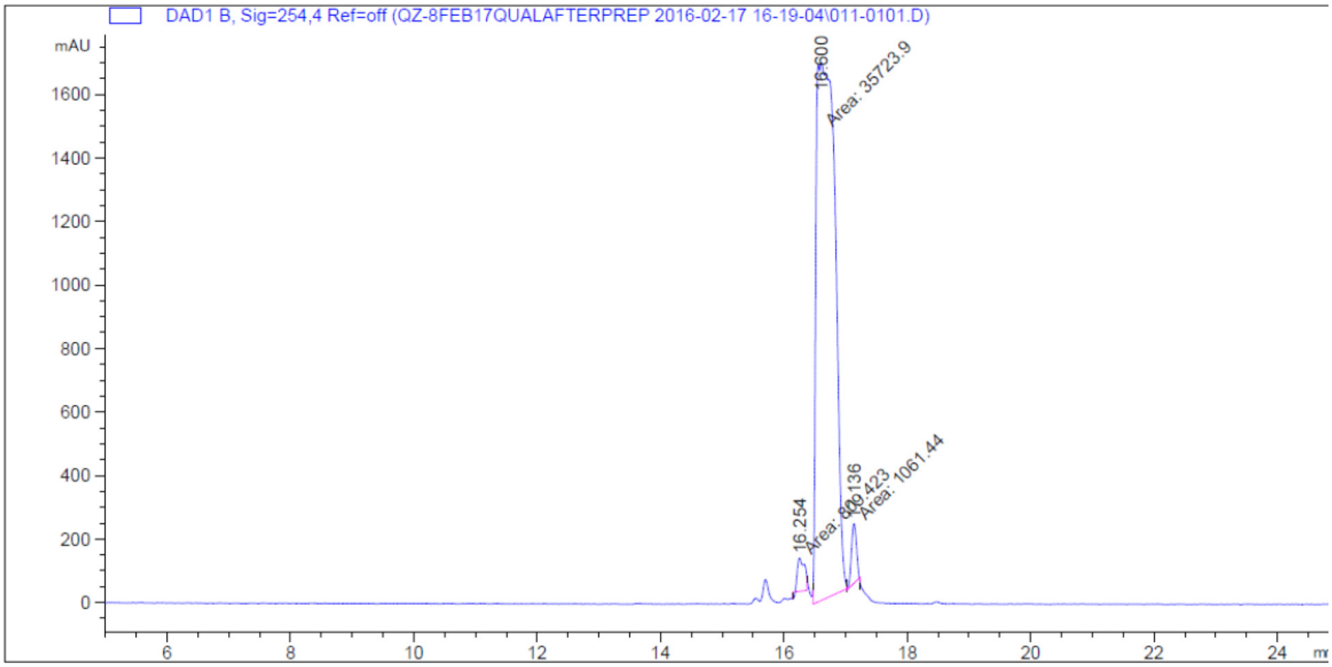


```
Signal 1: DAD1 B, Sig=254,4 Ref=off

Peak RetTime Type  Width     Area      Height     Area
  #   [min]        [min]   [mAU*s]     [mAU]        %
----|-------|----|-------|----------|----------|--------|
   1  16.254 MM T  0.1279  809.42334  105.45708   2.1530
   2  16.600 MM T  0.3513 3.57239e4  1694.89990  95.0236
   3  17.136 MM    0.0941 1061.43896  188.08916   2.8234

Totals :                3.75948e4  1988.44614

=====================================================================
                        *** End of Report ***
```

14

1H NMR MeOD 400 MHz

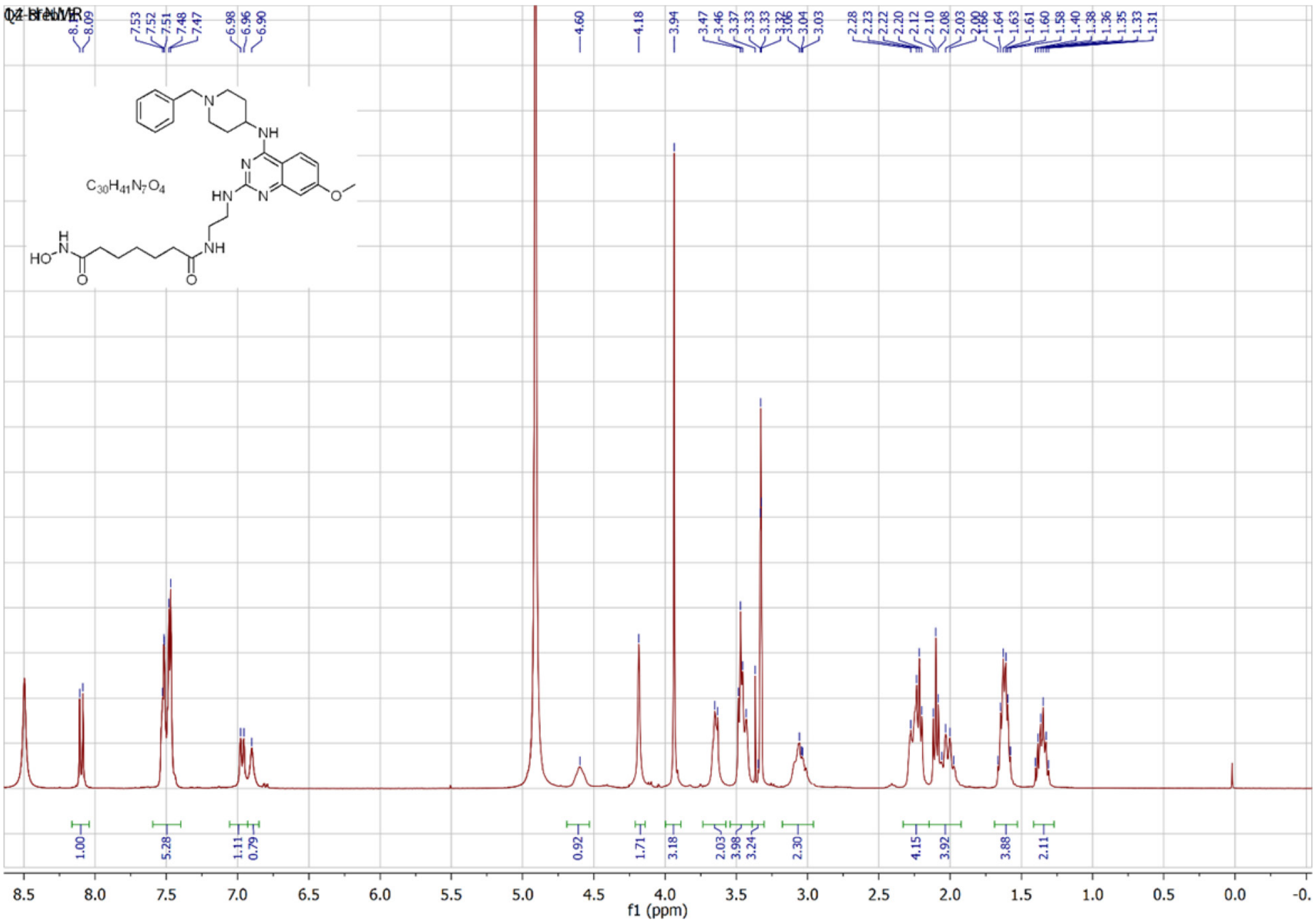

14
^{13}C NMR MeOD 100 MHz

^{13}C NMR MeOD 100 MHz

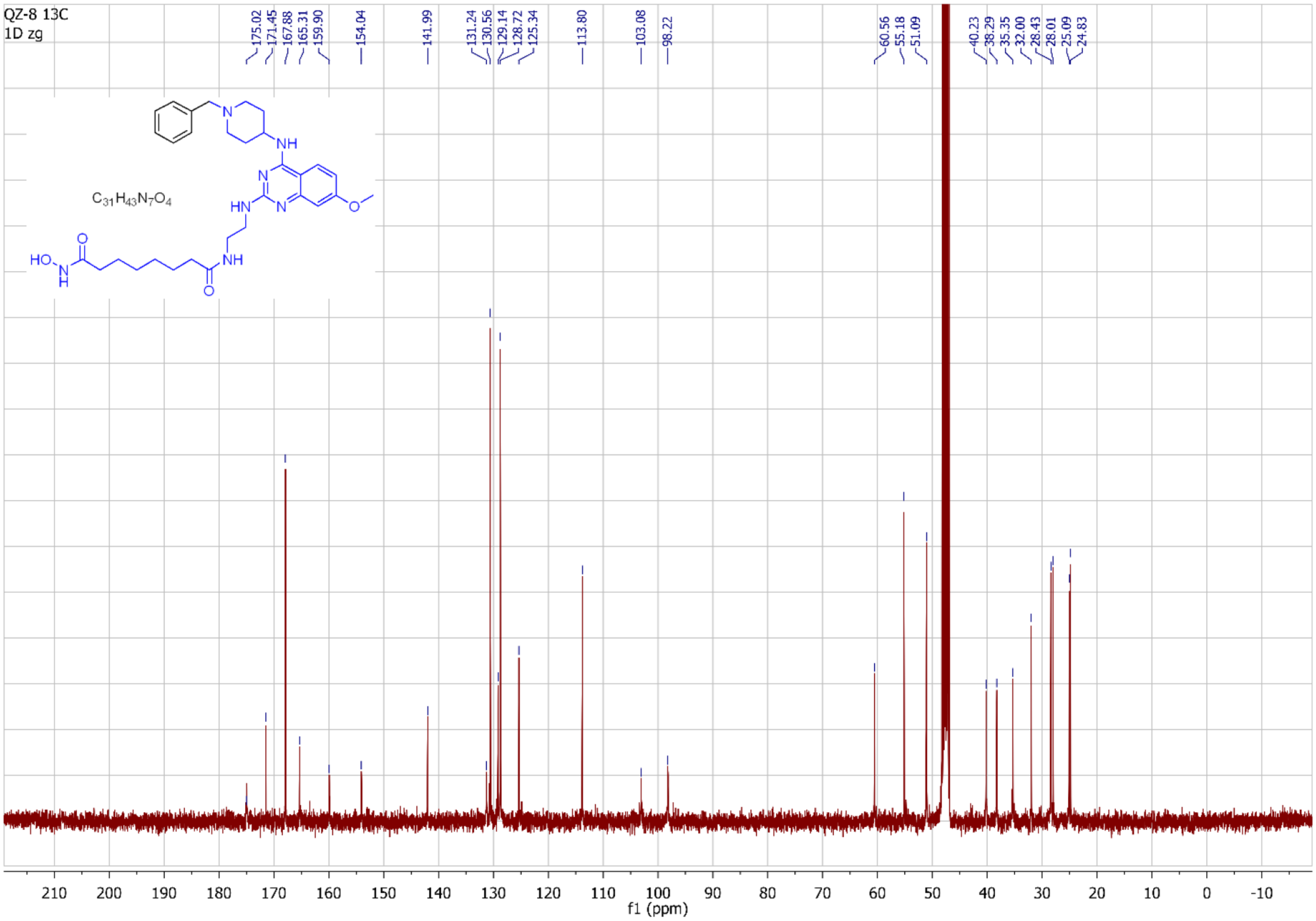

5b
1H NMR MeOD 400 MHz

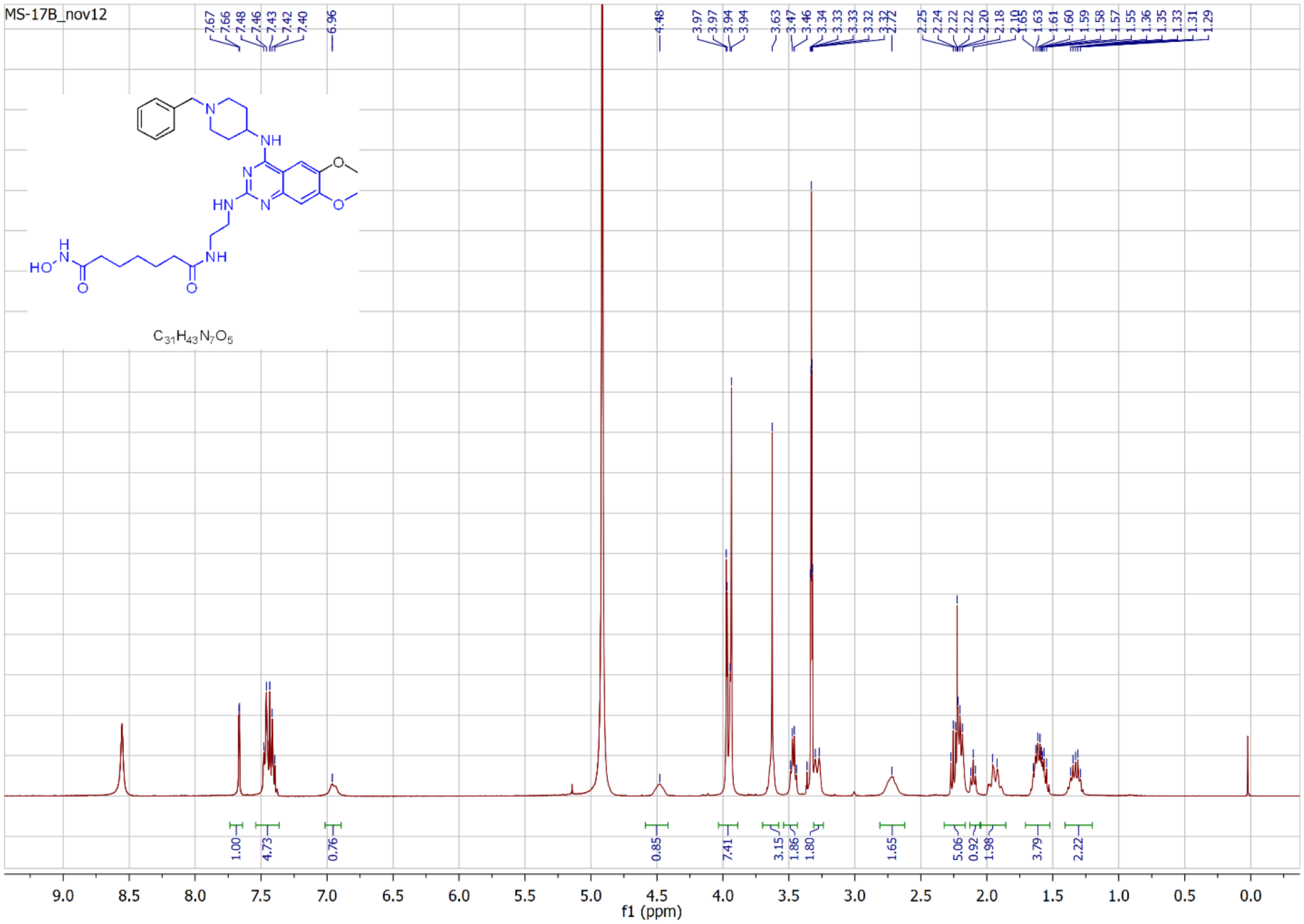

5b

^{13}C NMR MeOD 100 MHz

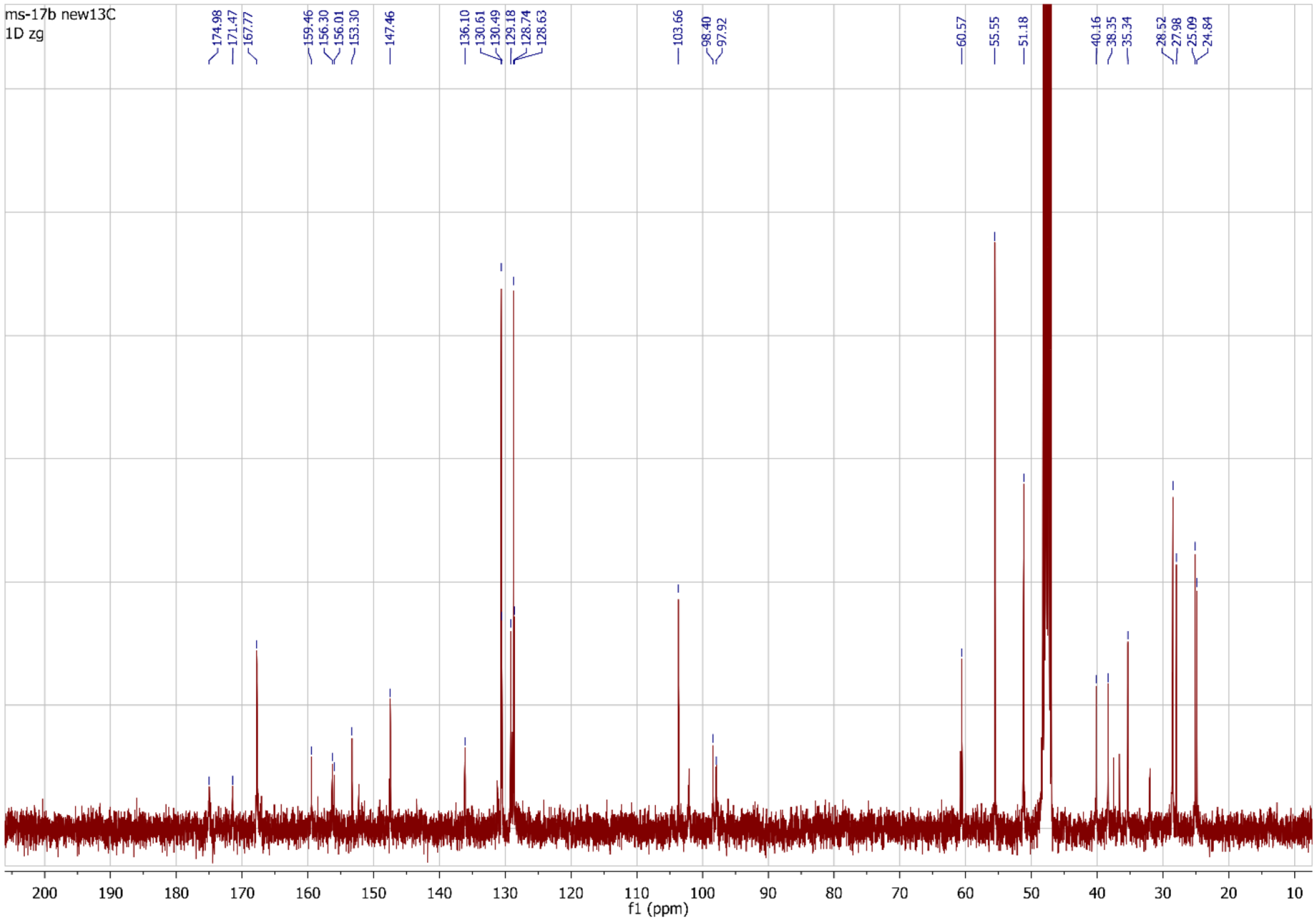

20
^{1}H NMR MeOD 400 MHz

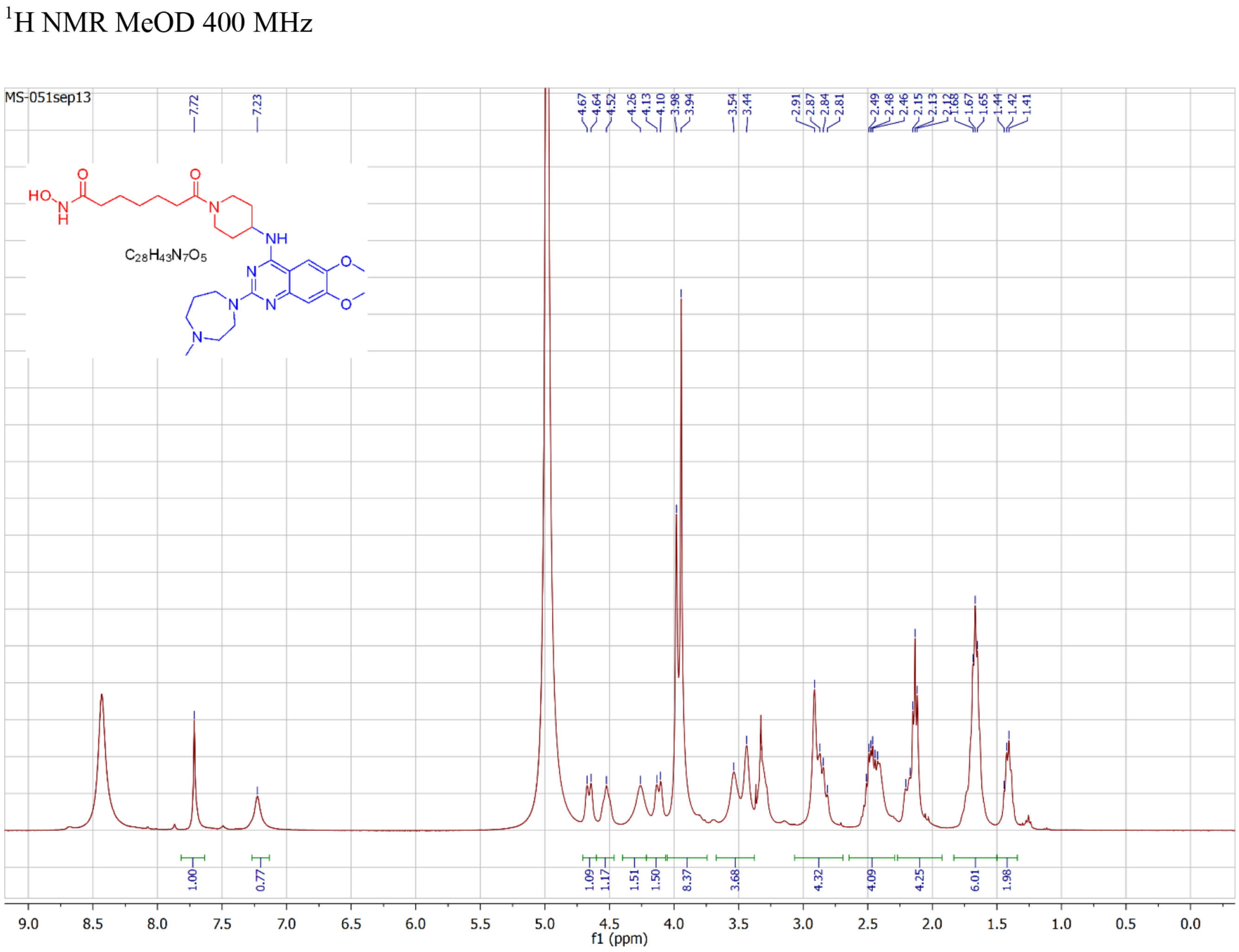

20

^{13}C NMR, MeOD 100 MHz

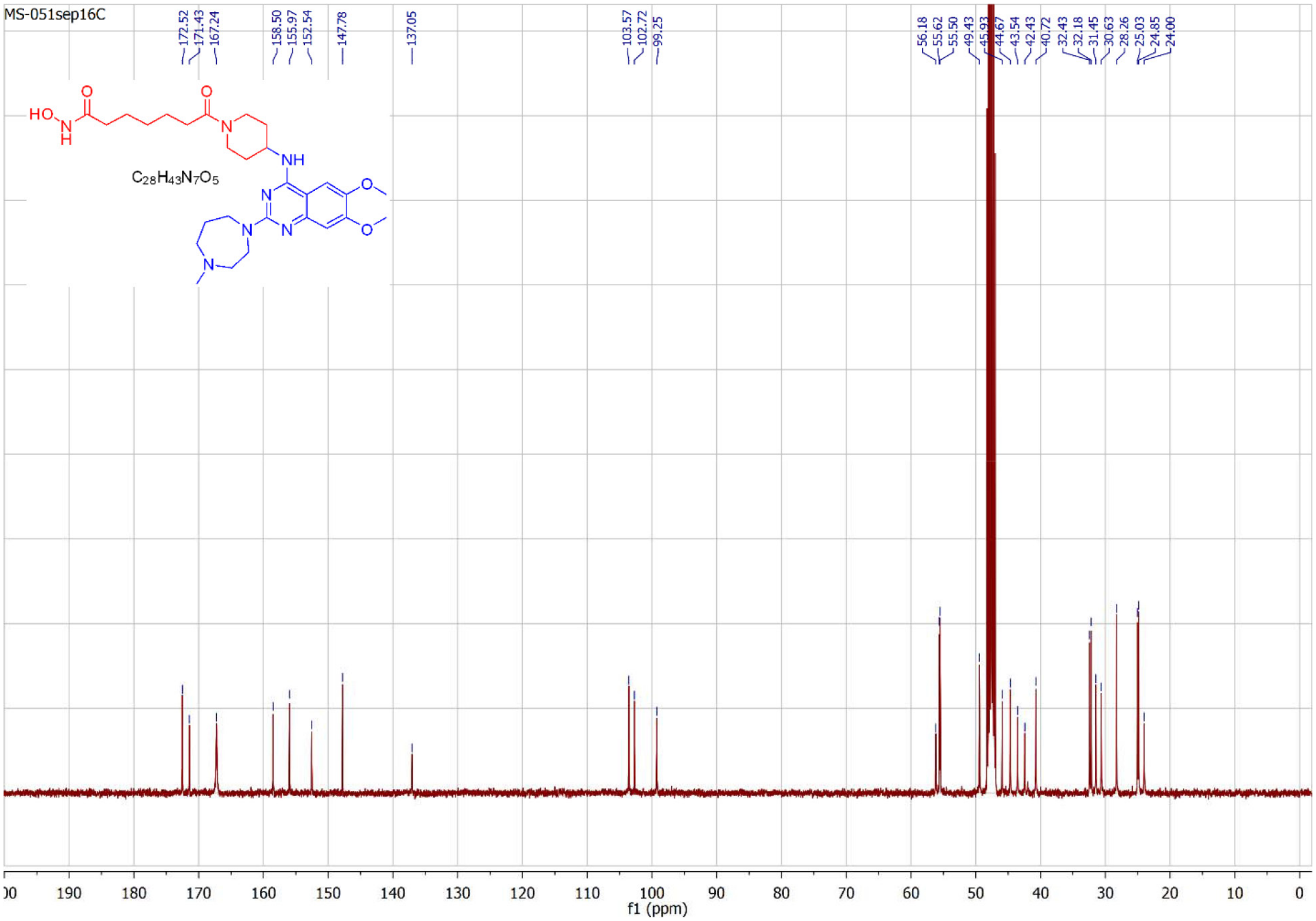

21

^{1}H NMR MeOD 400 MHz

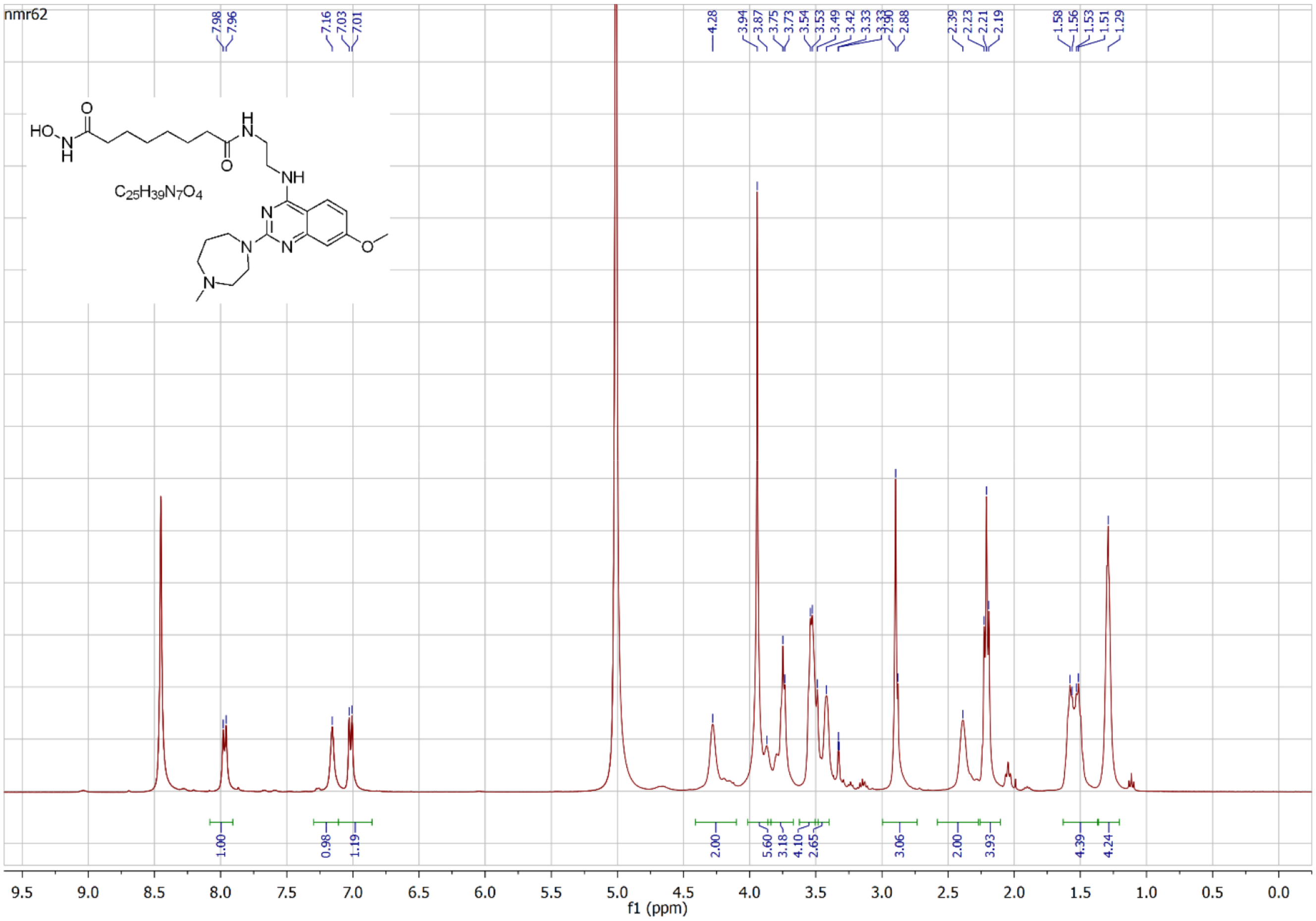

21

^{13}C NMR MeOD 100 MHz

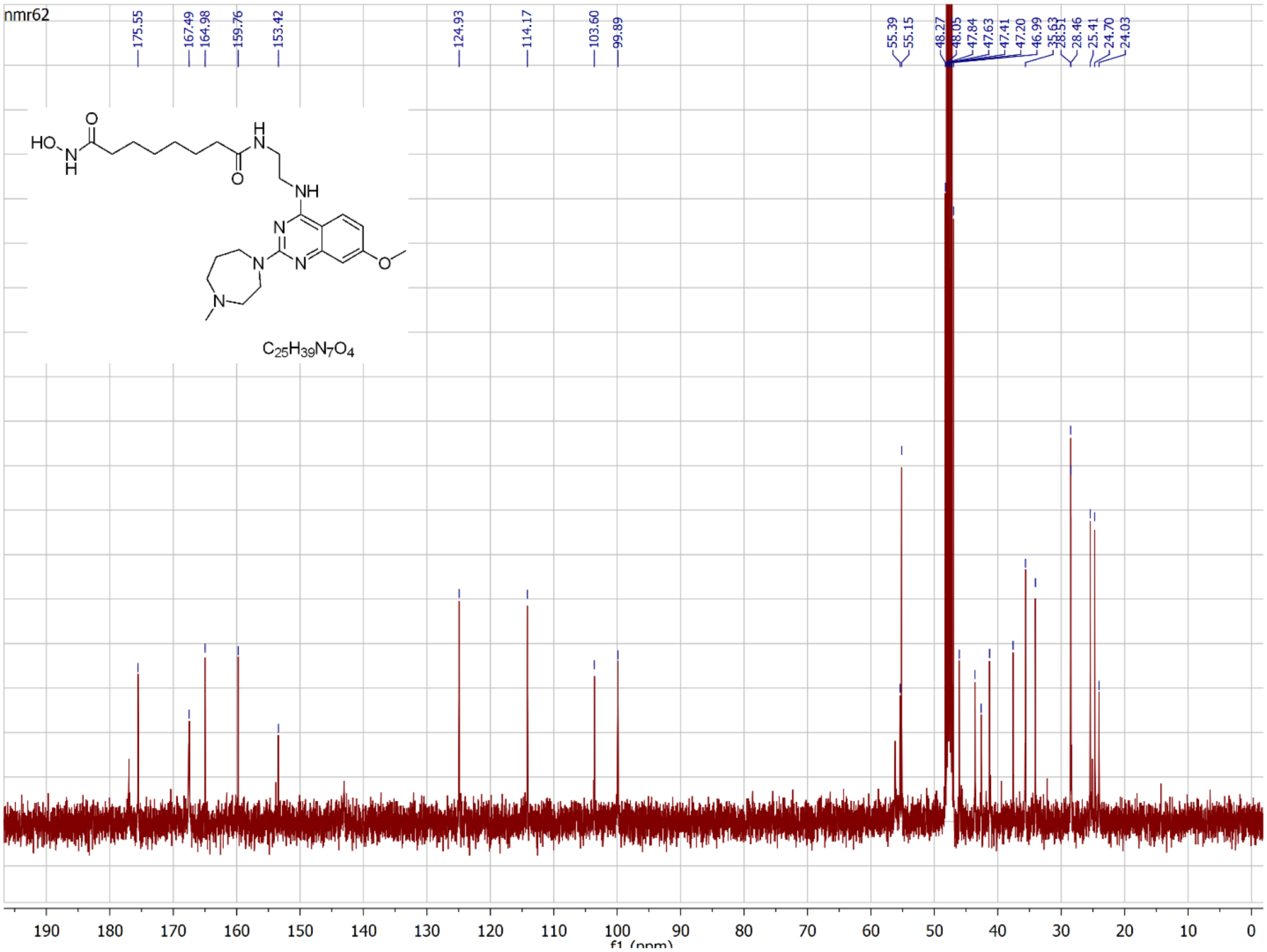

Supplement: Supplementary file 1 [file oncotarget-08-63187-s001.pdf]
